# Anaplastic large cell lymphoma arises in thymocytes and requires transient TCR expression for thymic egress

**DOI:** 10.1038/ncomms10087

**Published:** 2016-01-12

**Authors:** Tim I. M. Malcolm, Patrick Villarese, Camilla J. Fairbairn, Laurence Lamant, Amélie Trinquand, C. Elizabeth Hook, G. A. Amos Burke, Laurence Brugières, Katherine Hughes, Dominique Payet, Olaf Merkel, Ana-Iris Schiefer, Ibraheem Ashankyty, Shahid Mian, Mariusz Wasik, Martin Turner, Lukas Kenner, Vahid Asnafi, Elizabeth Macintyre, Suzanne D. Turner

**Affiliations:** 1 Department of Pathology, University of Cambridge, Cambridge CB21QP, UK; 2 Hematology and INSERM1151, Institut Necker-Enfants Malades, Université Sorbonne Paris Cité at Descartes and Assistance Publique-Hôpitaux de Paris, Paris 75743, France; 3 Institut Universitaire de Cancérologie Oncopole, 1 Avenue Irène Joliot-Curie, Toulouse 31059, France; 4 Department of Histopathology and Cytology, Addenbrooke's Hospital, Cambridge CB20QQ, UK; 5 Department of Paediatric Oncology, Addenbrooke's Hospital, Cambridge CB20QQ, UK; 6 Département de Cancérologie de l'Enfant et l'Adolescent, Gustave Roussy, 114 rue Edouard Vaillant, Villejuif 94805, France; 7 Department of Veterinary Medicine, University of Cambridge, Cambridge CB3OES, UK; 8 Centre d'Immunologie de Marseille Luminy (CIML), INSERM UMR1104, CNRS UMR7280, Aix-Marseille Université UM2, Marseille 13288, France; 9 Clinical Institute of Pathology, Medical University of Vienna, Vienna A-1090, Austria; 10 Molecular Diagnostics and Personalized Therapeutics Unit, College of Applied Medical Sciences University of Ha'il, Ha'il, Kingdom of Saudi Arabia; 11 Department of Pathology and Laboratory Medicine, University of Pennsylvania, Philadelphia, Pennsylvania 19104-6100, USA; 12 The Babraham Institute, Cambridge CB223AT, UK; 13 Ludwig Boltzmann Institute for Cancer Research (LBI-CR), Wahringerstrasse 13A, A-1090 Vienna, Austria; 14 Department of Laboratory Animal Pathology, University of Veterinary Medicine Vienna, 1210 Vienna, Austria

## Abstract

Anaplastic large cell lymphoma (ALCL) is a peripheral T-cell lymphoma presenting mostly in children and young adults. The natural progression of this disease is largely unknown as is the identity of its true cell of origin. Here we present a model of peripheral ALCL pathogenesis where the malignancy is initiated in early thymocytes, before T-cell receptor (TCR) β-rearrangement, which is bypassed in CD4/NPM–ALK transgenic mice following Notch1 expression. However, we find that a TCR is required for thymic egress and development of peripheral murine tumours, yet this TCR must be downregulated for T-cell lymphomagenesis. In keeping with this, clonal TCR rearrangements in human ALCL are predominantly in-frame, but often aberrant, with clonal TCRα but no comparable clonal TCRβ rearrangement, yielding events that would not normally be permissive for survival during thymic development. Children affected by ALCL may thus harbour thymic lymphoma-initiating cells capable of seeding relapse after chemotherapy.

## Abstract (web summary)

Anaplastic large cell lymphoma is characterized by an NPM–ALK fusion but the cell of origin for this cancer is unclear. Here, the authors show that, in an NPM–ALK mouse model, the tumours likely arise from early thmyocytes and require an initial burst of TCR signalling for initiation.

## Graphical abstract

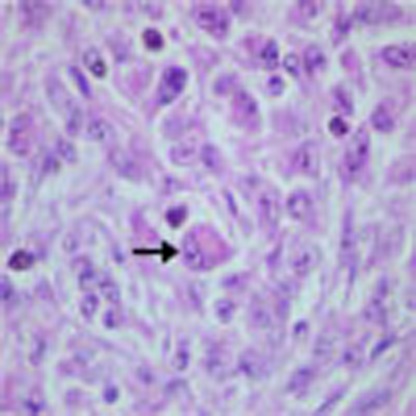

Nucleophosmin-anaplastic lymphoma kinase (NPM–ALK) positive anaplastic large cell lymphoma (ALCL) is considered as a peripheral T-cell lymphoma and is associated with the *t*(2;5)(p23;q35) translocation, identified to be the fusion of a kinase gene, *ALK*, to a nucleolar protein gene, *NPM1* (ref. 1). ALCL is defined by expression of the CD30 marker of activated B and T cells and, in some cases, T cell markers such as CD4 or CD8, as well as production of cytotoxic molecules such as perforin, which together with the presence of clonal T-cell receptor (TCR) rearrangements, suggests an activated T-cell origin^2^,^3^,^4^,^5^,^6^. Other studies have proposed a Treg cellular origin (owing to the generation of FoxP3 transcripts and production of IL10 and TGFβ by ALCL cell lines) or more recently a Th17 phenotype (owing to production of IL17 by tumour cells)^5^,^6^,^7^,^8^. Whether these cellular attributes are a remnant of the cell of origin or are NPM–ALK induced remains to be fully elucidated. Despite the presumed T-cell affiliation, ALK^+^ ALCL rarely express TCRβ or CD3ɛ by immunohistochemistry^9^, and the CD3 signalling cascade is downregulated in an NPM–ALK-dependent manner by transcriptional and epigenetic modification^10^. More recently, we have shown that a subpopulation of ALCL cancer stem cells (CSC) express genes enriched within the gene set of early thymic progenitors (ETPs), hinting towards a primitive cell of origin^11^. Our data suggest that the seeds of ALCL are sown in the thymus, or even earlier in hemopoietic development, perhaps indicating that events in the thymus, or once the primed cells exit the thymus, might shape the final cell phenotype. To investigate this hypothesis, we carried out a thorough analysis of TCR rearrangements in human ALCL to look for evidence of NPM–ALK subverted thymic T-cell development. During normal T-cell development, TCRα VJ rearrangement is preceded by TCRβ VDJ rearrangement and massive cellular expansion of thymocytes during pre-TCR β-selection. The process of β-selection is driven by the pre-TCR composed of a pre-TCRα expressed at the cell surface together with the TCRβ chain. TCR immunogenetic profiles by multiplex PCR can therefore be used to identify lineage affiliation and stage of maturation arrest.

Our data show that most ALK^+^ ALCL demonstrate immunogenetic evidence of in-frame TCR rearrangement, 25% displaying atypical TCR profiles not normally selected for during (or permissive of) TCR-αβ lineage thymic development. In normal T-cell ontogeny, these events would result in a failure of β-selection and thymic cell death, suggesting that event(s) in the thymi of these patients had subverted β-selection. We therefore used the CD4/NPM–ALK (CD4NA) thymic T cell lymphoma model^12^ to investigate the potential contribution of recombinase activating gene (Rag)-mediated TCR rearrangement and TCR signalling to the natural progression of NPM–ALK-driven T-cell lymphoma. In the absence of Rag-mediated TCR rearrangements, NPM–ALK can induce ‘T cell maturation' to the CD4^+^CD8^+^ double-positive (DP) and CD4 single-positive (SP) stages of T-cell development, allowing the formation of thymic lymphomas. We hypothesized that expression of some form of TCR might be required to initiate thymic egress and therefore backcrossed the CD4NA transgenic line to the class I-restricted Ova-specific TCR transgenic line OT1, on both a Rag-competent and Rag-deficient background^13^,^14^. The presence of the OTI TCR moves the site of lymphoma presentation to the periphery although tumour cells do not express a TCR, and TCR stimulation prevents lymphoid oncogenesis; as such this model more resembles human ALCL, which also rarely express a TCR^9^,^10^. These data suggest that peripheral ALK^+^ ALCL depends on transient expression of a functional TCR to enable thymic emigration of primed T lymphocytes and its subsequent downregulation once cells are in the periphery to permit transformation.

## Results

### Abnormal TCR rearrangements in human ALCL tumours

**Figure 1:**
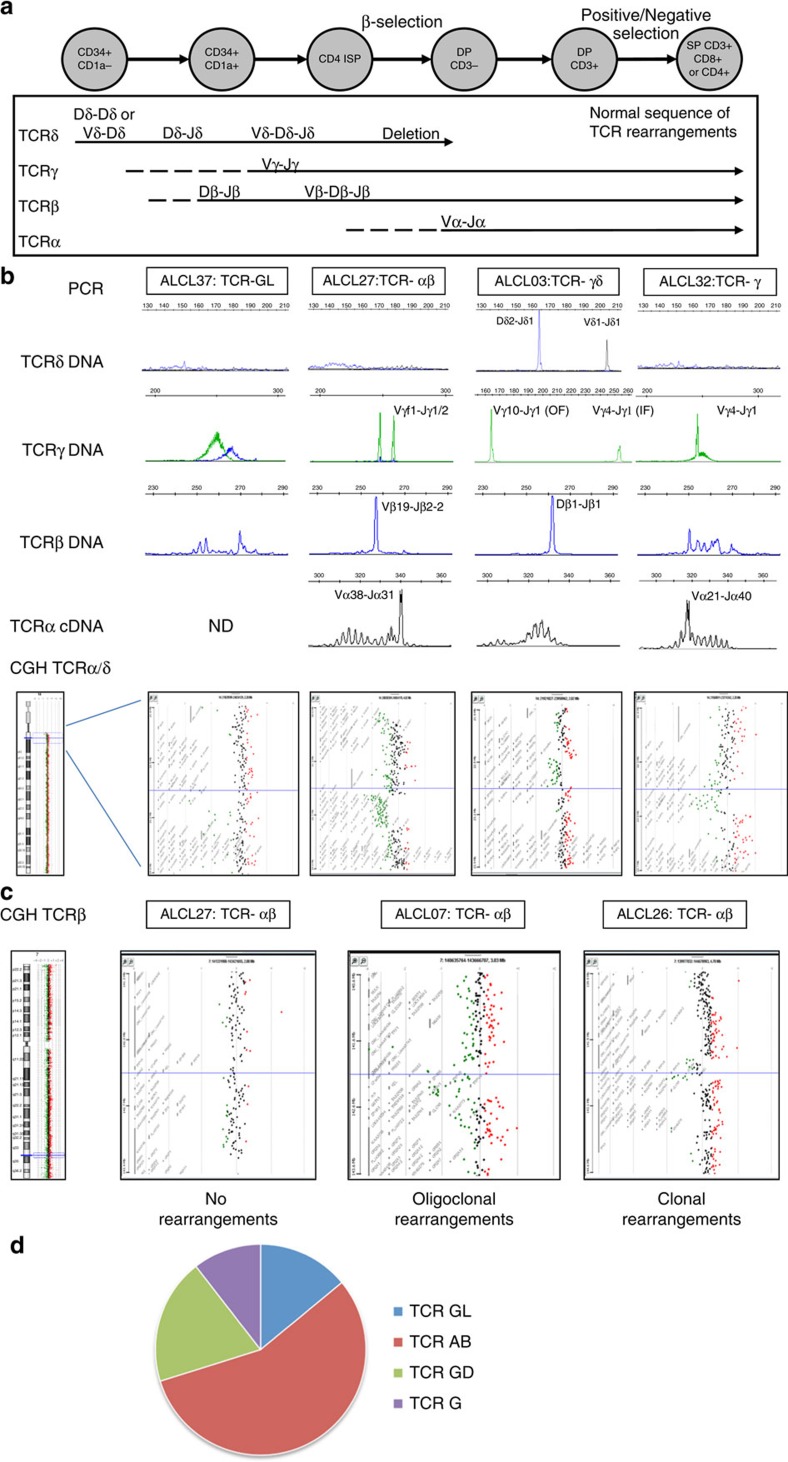
Representative TCR rearrangements in ALCL as detected by Gene Scan PCR and CGH. (**a**) During normal T cell development, rearrangement of TCR genes occurs in a temporal manner whereby rearrangement of δ precedes γ, which is followed by β and then α, the latter of which coincides with deletion of the δ locus. DP, double positive; ISP, intermediate single positive; SP, single positive. (**b**) Typical PCR from DNA (γ, β, δ) or RNA (α) depicting the presence/absence and type of clonal rearrangements for each of the four TCR categories identified are shown, as are Agilent 180K CGH TCRα/δ profiles. (**c**) Three examples representing the range of CGH TCRβ V-J rearrangements observed among the TCRαβ cases: absent (12 out of 17), oligoclonal (2 out of 17) or clonal (3 out of 17). (**d**) The percentage of human ALCL categorized as having either TCR germ line (GL), TCRαβ (TCR AB), TCRγδ (TCR GD) or TCRγ only (TCR G) rearrangements. ND, not determined.

As NPM expression is ubiquitous, the NPM–ALK fusion could lead to expression of ALK at all stages of thymic development^15^. To investigate whether ALCL cells have passed through the thymus while expressing NPM–ALK, we analysed the status of TCR rearrangements in human tumours. TCRδ, TCRγ and TCRβ genes were amplified from DNA of ALCL tumours, and clonal populations identified by genescan analysis of PCR products, with an approximate sensitivity of 1–5% (Fig. 1, Table 1, Supplementary Table 1 and summarized Supplementary Fig. 1). To refine the TCR status, 180K Agilent CGH array analysis and PCR detection of clonal TCRα transcripts from complementary DNA (cDNA) using Vα and Cα primers, was performed for selected cases (Table 1).

A total of 57 ALK^+^ ALCL with at least 40% tumour cell content were analysed. T lymphoid immunohistology and cytotoxic status is summarized in Table 1 and detailed in Supplementary Table 1. Overall, clonal TCRγ VJ rearrangements were identified by PCR in 49 (75%), TCRβ VDJ in 33 (58%) and TCRδ VDJ in 11 (19%) cases (Table 1). On the basis of these data, we classified the ALCL into four distinct categories based on the TCR rearrangement profile observed: TCR germline (GL), TCRαβ, TCRγδ and TCRγ only.

Eight (14%) tumours demonstrated no significant evidence of clonal TCR rearrangement by PCR or TCRα/δ CGH, despite at least 50% tumour cell content (Fig. 1b,d, Table 1 and Supplementary Table 1) and were categorized as TCR GL. As such, they represent the molecular equivalent of null ALCL (although six out of eight cases expressed cell surface markers associated with a T-cell phenotype (Supplementary Table 1)).

Tumours were classified as TCRαβ if a clonal TCRβ VDJ rearrangement was detected by PCR on at least one allele, in the absence of TCRδ VDJ rearrangement, consistent with bilateral TCRδ deletion during TCRα rearrangement. This was the predominant category (32 out of 57; 56%; Fig. 1d). The vast majority (16 out of 17 tested) of TCR-αβ ALK^+^ ALCL demonstrated clear evidence of TCRα VJ on at least one allele by CGH and clonal TCRα transcripts were identified by reverse-transcriptase PCR (RT–PCR) in 10 out of 15 CGH-positive cases (Fig. 1b). Unexpectedly, TCRβ rearrangements, despite being clearly detected by PCR, were only detected by CGH in a minority of cases (3 out of 17, 18%), with two additional cases (ALCL19 and 07) demonstrating heterogeneous deletions (Fig. 1c). This was not owing to insufficient resolution of the Agilent 180K arrays, as even TCRβ rearrangements involving 5′ Vβ were not seen and repeat CGH using Affymetrics high density Cytoscan arrays (2.7 M probes) in three TCRβ PCR VDJ+/CGH− TCRαβ cases confirmed clear evidence of TCRα VJ rearrangement, but no detectable TCRβ VDJ, with only one case demonstrating an expected clonal incomplete TCRβ DJ (ALCL24) (Supplementary Fig. 2).

Sequence analysis of clonal PCR products demonstrated at least one in-frame TCRα rearrangement in eight out of nine TCRαβ cases, with three cases demonstrating two in-frame rearrangements. Sequencing of the TCRβ VDJ rearrangements from 16 TCR-αβ ALCL (Supplementary Table 1) identified a mono-allelic, in-frame rearrangement in all but one case, ALCL 26, notably one of the rare cases to be positive by TCRβ CGH. Despite this evidence of a functional TCRαβ, none of the 16 ALCL analysed by immunohistochemistry demonstrated detectable TCRβ expression on tumour cells (Supplementary Fig. 3A). Thirteen TCRγ alleles were sequenced from eight TCRαβ cases, but as expected, only two (15%) alleles were in-frame (Supplementary Table 1).

Taken together, TCRαβ tumours demonstrate clear evidence of in-frame clonal TCRα rearrangement but only minor, in-frame TCRβ rearrangements which are detected by PCR but rarely by CGH array, in keeping with heterogeneous, on-going TCRβ rearrangements within the TCRα clone, or with reactive, tumour infiltrating lymphocytes. To determine whether these on-going rearrangements bore any clonal ancestry, we cloned and sequenced the TCRβ rearrangements from ALCL19, but none of the minor rearrangements shared any apparent ancestral TCRβ CDR3 DJ sequence.

Six cases demonstrated clonal TCRγ rearrangements but no TCRδ rearrangement and, at the most, incomplete TCRβ rearrangement (three monoallelic DJ), and were labelled TCRγ-only (Fig. 1b,d). Surprisingly, the TCRα locus was found to be rearranged in two out of four cases by CGH and clonal TCRα VJC transcripts were detected in five out of six by RT–PCR (Fig. 1b, Supplementary Fig. 2, ALCL30 and Table 1). Strikingly, all six TCRα rearrangements sequenced from these five ALCL were in-frame, despite the apparent absence of clonal TCRβ VDJ rearrangement. This was not due to false negative TCRβ PCR results, as 180K CGH analysis did not identify TCRβ rearrangement in four cases tested (Supplementary Table 1) and high resolution 2.7M Cytoscan analysis of ALCL30 only identified the expected TCRβ DJ (Supplementary Fig. 2B). Only one (ALCL23) had no evidence of TCRα rearrangement by RT–PCR and CGH. As such, it resembled a TCR GL ALCL with an isolated ‘immature' out-of-frame VγfI-JP1/2 TCRγ rearrangement. In the absence of TCRβ, TCRα could, at least hypothetically, pair with TCRγ, as all cases had undergone at least mono-allelic rearrangement, but only one of the six TCRγ alleles sequenced was in-frame. As such, five out of six TCRγ-only cases demonstrate potentially functional TCRα rearrangement in the absence of TCRβ rearrangement and only differ from TCRαβ cases by the absence of a TCRβ clonal VDJ peak by PCR.

Eleven cases (19%) were classified as TCR-γδ since clonal TCRγ VJ and TCRδ VDJ rearrangements were detected (Fig. 1b,d). This category demonstrated a uniform genotype, with 10 out of 11 demonstrating a Vδ1-Jδ1 /Dδ2-Jδ1 genotype and an in-frame TCRγ and TCRδ rearrangement on at least one allele (Supplementary Table 1). The 180K CGH profiles confirmed only TCRδ but no TCRα or TCRβ rearrangements in the six cases tested (Supplementary Table 1 and Table 1). They all demonstrated an incomplete TCRβ DJ on at least one allele (below the resolution of CGH detection). ALCL36 also demonstrated a complete, in-frame TCRβ VDJ rearrangement but was classified as a TCR-γδ since the TCRδ rearrangements were bilateral, the clonal TCRγ was in-frame and no TCRα transcripts were detected by RT–PCR. Thus, as for TCR-αβ ALCL, TCR-γδ ALCL have immunogenetic evidence of a potentially functional TCR.

In summary, PCR and CGH analysis of TCR rearrangements in ALK^+^ ALCL showed that two-thirds (TCR-αβ and TCRγ-only) demonstrate a major in-frame TCRα clonal rearrangement, which is not accompanied by a comparable major TCRβ clonal population but by minor TCRβ clonal populations that are predominantly in-frame and may originate within or independently to the TCRα clonal population and are not associated with detectable TCRβ protein by immunohistochemistry. The remaining third are divided between immunogenetic ‘Null-ALCL' (TCR germline, 14%) and immunogenetic TCR-γδ ALCL (19%).

We therefore hypothesized that, in keeping with our prior suggestion that NPM–ALK may replace the TCRβ signalling cascade^16^, NPM–ALK may allow an immature hematopoietic cell to bypass thymic β-selection to a stage when TCRα VJ rearrangement becomes possible, thus enabling abnormal T lymphoid differentiation and implying a thymic origin to at least a proportion of ALCL. To evaluate whether ongoing recombinase activity could occur in established ALK^+^ ALCL tumours, we looked for *RAG1* transcripts in 52 cases, but all were negative, as were all the three ALCL cell lines tested in contrast to the immature T-ALL cell lines arrested at a thymic stage of development (Supplementary Fig. 3B).

### NPM–ALK can induce thymic ‘T cell maturation'

**Figure 2:**
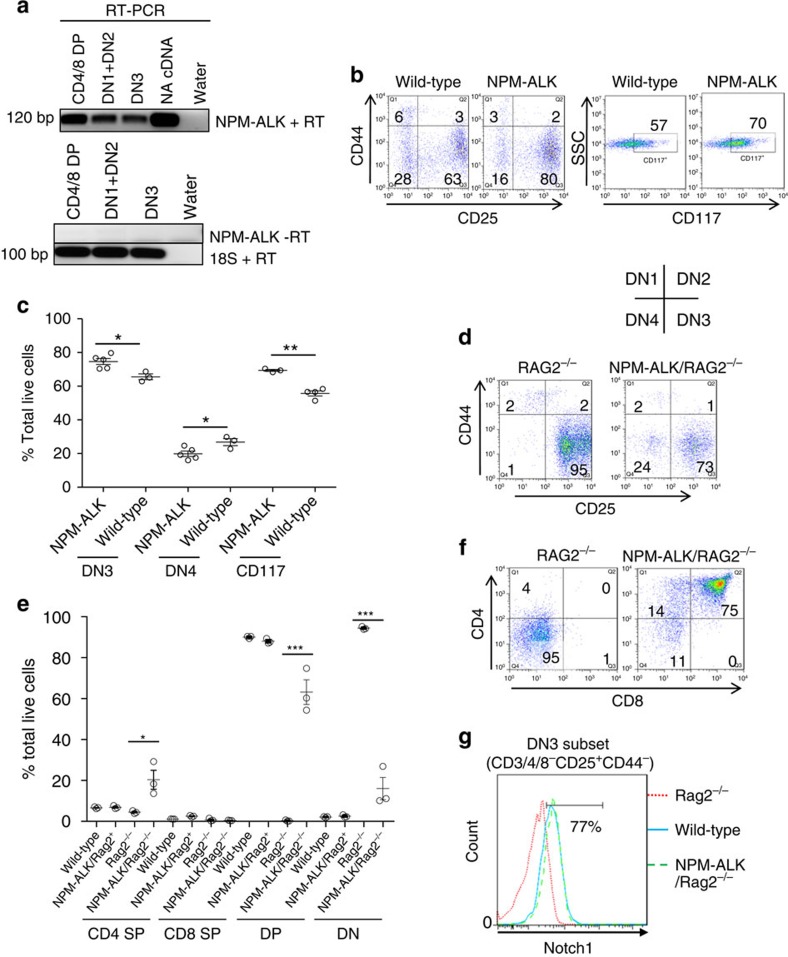
NPM–ALK induces a partial DN3 block which is overcome in the absence of RAG. (**a**) CD4NA transgenic mice thymi were sorted into the T-cell subsets indicated and extracted RNA was assessed for the presence of NPM–ALK transcripts. SP, single positive; DP, double positive; DN, double negative; RT, reverse transcription. (**b**) The thymi (total thymic cell count comparing wild-type with transgenic mice is equal at this age) of 5-week-old CD4NA transgenic mice were analysed for the percentage of cells in each stage of the double-negative stages (gated on CD3/4/8^−^ cells) of thymic T-cell development. A representative example of five mice analysed is shown. Numbers in the graphs represent the percentage of the total DN pool. (**c**) An overview of the percentage of cells at each stage of thymic T-cell development in groups of five mice of each of the indicated genotypes at 5 weeks of age. Data represent individual mouse counts and the mean value, *P*=0.0368, *P*=0.015 and *P*=0.0007 for the DN3, DN4 and CD117 subpopulations, respectively (Student's *t*-test assuming equal variance). (**d**) Thymic development at the double-negative stages was assessed in the indicated murine genotypes by flow cytometry. A representative example of three mice analysed is shown and numbers in the graphs represent the percentage of the DN pool. (**e**) An overview of the percentage of cells at each stage of thymic development in the indicated genotypes at 9 weeks of age. Data represent individual mouse counts and the mean, error bars indicate the standard deviation, *P*=0.0267, *P*=0.0005 and *P*=0.0001 for the CD4SP, DP and DN subpopulations, respectively, *n*=10 of each genotype, Student's *t*-test. (**f**) NPM–ALK restores thymic development in RAG2^−/−^ mice. A representative example of three mice analysed is shown and numbers represent the percentage of the total live thymocyte count. (**g**) NPM–ALK expression in the thymus restores Notch1 expression on the surface of DN3 thymocytes in RAG2^−/−^ mice. A representative example of three mice analysed of each of the indicated genotypes is shown with the positive gate drawn according to the isotype control.

To elucidate whether NPM–ALK expressing thymocytes might develop and eventually undergo transformation in the absence of a functional TCR rearrangement, we generated transgenic mice expressing NPM–ALK at all stages of thymic development which lacked the *rag2* gene (Fig. 2a). RAG2 is required for TCR rearrangement hence *rag*2^−/−^ mice have small thymi devoid of mature T cells but contain normal numbers of T-cell progenitors blocked in development at the DN3 stage. We analysed the thymi of both CD4NA/RAG^+/+^ and CD4NA/RAG2^−/−^ mice before the presentation of overt tumours. In CD4NA/RAG^+/+^ mice at 5 weeks of age, there is an accumulation of phenotypic DN3 cells. (Fig. 2b,c, absolute cell counts are shown in Supplementary Table 2). There was also an increase in the percentage of the total thymic population expressing the cell surface marker CD117, a protein whose expression is switched off at the DN3/4 stage of thymic development (Fig. 2b,c). These data suggest a delay in T-cell development at this stage induced by NPM–ALK activity and consistent with the time at which β-selection occurs.

In the CD4NA/RAG2^−/−^ mice, the cellularity of the thymus was increased significantly over numbers seen in RAG2^−/−^ mice but not to the levels observed in wild-type mice (Fig. 2e, absolute cell counts shown in Supplementary Table 3). Cell numbers observed in the RAG2^−/−^ line are consistent with previous reports^13^. In CD4NA/RAG2^−/−^ mice, the numbers of DN3 cells were over-represented compared with wild type mice. Notably, the majority of thymocytes in CD4NA/RAG2^−/−^ were DP (Fig. 2e,f). These data indicate NPM–ALK can promote the bypass of the β-selection checkpoint. In further support of these data, 32% less DN4 cells isolated from CD4NA mice express TCRβ on the cell surface compared with wild-type littermate control mice (Supplementary Fig. 4A).

Furthermore, DN3 cells isolated from the CD4NA/RAG2^−/−^ transgenic mice expressed cell surface Notch1, consistent with apparent maturation of thymocytes beyond the DN3 stage (Fig. 2g) as well as upregulation of the nutrient transporters CD98 and CD71 required to meet the metabolic demands of developing thymocytes (Supplementary Fig. 4B). Indeed, CD98 and CD71 upregulation are usually only seen in the presence of signalling by Notch1 and the pre-TCR, suggesting that NPM–ALK not only induces Notch1 expression but also compensates for absence of the pre-TCR^17^.

### The CD44^hi^ population is the precursor of tumour growth

An abnormal population of thymocytes is detectable in CD4NA transgenic mice at later ages, when thymic cellularity is still within the normal range. CD4/CD8 mature T cells express the immature cell surface marker CD117 as well as CD44, the latter being normally restricted to DN1/2 thymocytes (Supplementary Fig. 5A). Whether the increase in CD44^hi^ cells is a remnant of expression on DN1/2 cells or NPM–ALK induced upregulation of CD44 is unclear, though the latter explanation is supported by experiments in which NPM–ALK is capable of driving expression of luciferase from the CD44 promoter (Supplementary Fig. 5B).

These data suggest that NPM–ALK drives asynchronous TCRβ independent proliferation of DN3-like cells and the maturation to DP cells, without loss of DN1/2 characteristics such as expression of CD44 and CD117. This may provide an oncogenic advantage to this population, which is not subjected to thymic TCR-mediated selection. Indeed, this CD44^+^ population gave rise to thymic tumours when transplanted intravenously into recipient mice, again consistent with an origin in ETPs in this model system (Supplementary Fig. 5C).

### NPM–ALK drives TCR-independent thymic tumour formation

**Figure 3:**
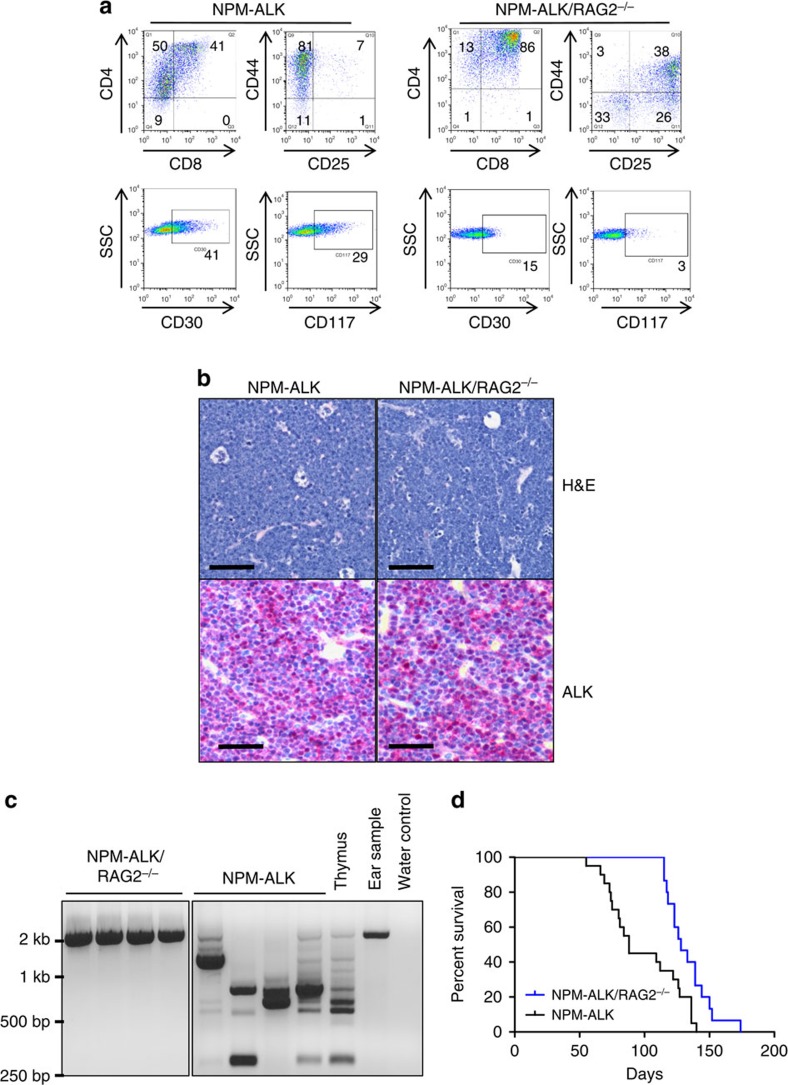
Tumours reminiscent of cortical thymocytes develop in NPM–ALK transgenic mice in the absence of RAG. (**a**) The phenotype of thymic tumours presenting in CD4NA and CD4NA/RAG^−/−^ mice are reminiscent of cortical thymocytes. The numbers in the graphs represent the percentage of the total live tumour cell count. These data are representative of at least 10 tumours analysed. (**b**) Typical histopathological presentation of the tumours which are positive for ALK expression, × 400 magnification. Scale bar, 50 μm. (**c**) TCRβ rearrangements in the indicated murine tumour genotypes compared with normal thymus and non-lymphoid DNA; four representative examples of each are shown. (**d**) Kaplan–Meier survival curve for the CD4NA and CD4NA/RAG2^−/−^ mice, *P*<0.005, log rank test. NPM–ALK/RAG2^−/−^, *n*=20; NPM–ALK, *n*=15.

Despite the absence of TCR rearrangement, the CD4NA/RAG2^−/−^ mice produce thymic tumours consisting of cells reminiscent of the double-positive stage of T-cell development (Fig. 3a, Table 2). However, tumours arising in the CD4NA or CD4NA/RAG2^−/−^ lines display the same histopathology, consisting of NPM–ALK-expressing diffuse, homogenous sheets of medium-sized cells with complete effacement of the normal thymic architecture (Fig. 3b). As expected, the CD4NA/RAG2^−/−^ tumours, despite expressing CD4±CD8 do not display TCR rearrangements (Fig. 3c). These data demonstrate that TCR rearrangement is not necessary for cellular transformation in the thymus, although the development of tumours in CDNA/RAG2^−/−^ mice compared with CD4NA is delayed, suggesting that this process may enhance tumorigenesis (Fig. 3d). NA in the absence of TCR rearrangement therefore allows asynchronous thymic development to the CD4/8 DP stage and development of cortical thymic lymphoma.

Murine tumours are always restricted to the thymus, whereas ALCL in humans present for the most part in the periphery and are often extranodal (although mediastinal involvement has been observed in as many as 50% of human cases^18^). It is therefore clear that if the tumours initiate in the thymus, something must contribute to thymic egress. During normal thymic maturation, this is normally provided by surface expression of a TCR/CD3 complex. ALCL is, in contrast, characterized in both humans^9^ and our murine CD4NA/RAG2^−/−^ model by complete absence of a TCR/CD3 complex, thus potentially explaining the failure to exit the thymus, at least in mice. This is not always the case, as CD4NA mice can, in rare cases, present with peripheral tumours (in our hands one out of 50 CD4NAmice develop tumours in the peripheral lymph nodes). In addition, our data show that the majority of human ALCL demonstrate immunogenetic evidence of an in-frame TCRαβ or TCRγδ, suggesting prior expression of a functional TCR. We therefore restored TCR expression in the mouse model by introducing a TCR transgene.

### Presence of an OT1 TCR results in peripheral T-cell lymphoma

**Figure 4:**
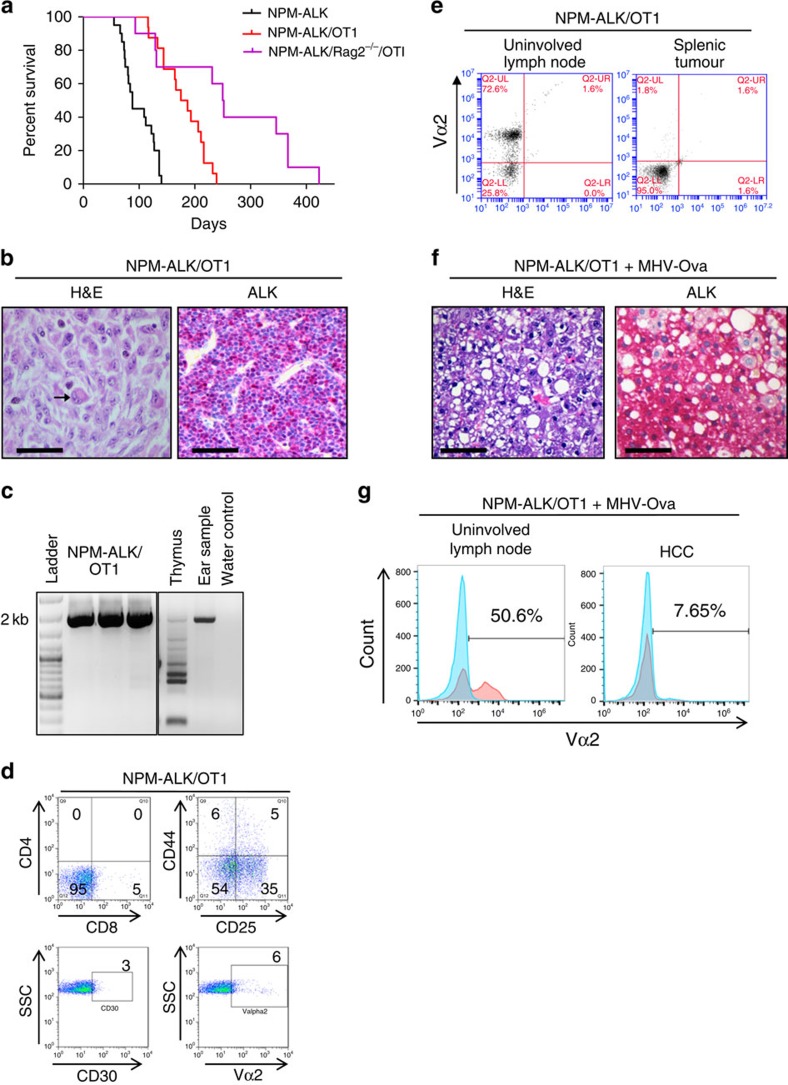
CD4NA/OT1 mice develop peripheral T-cell lymphomas only in the presence of RAG and more closely mimicking human ALCL. (**a**) Kaplan–Meier survival curve, CD4NA *n*=20, CD4NA/OT1 *n*=16, CD4NA/OT1/RAG2^−/−^ *n*=10, *P*=0.0052 (CD4NA/OTI versus CD4NA/OTI/RAG2^−/−^), log rank test. (**b**) A typical histopathological presentation of the tumours presenting in the CD4NA/OTI mouse line. A cell with morphological similarities to a ‘hallmark' cell is indicated by the arrow, × 400 magnification; scale bar, 50 μm. (**c**) Endogenous TCRβ rearrangements in tumours that present in the CD4NA/OTI mouse line, three representative examples are shown. (**d**) The majority of tumours that develop in the mice are of a ‘null' cell phenotype lacking expression of all T cell defining cell surface markers including the transgenic OT1 TCR (Vα2). Numbers within the graphs represent the percentage of the total live tumour cell count. (**e**) A representative example of a null cell tumour arising in the spleen not expressing the Vα2 TCR (right panel), whereas healthy lymph nodes taken from the same mouse do express the Vα2 TCR (left panel). (**f**) CD4NA/OTI mice administered MHV-Ova develop hepatocellular carcinoma, × 400 magnification; scale bar, 50 μm. (**g**) HCC tumours do not express the OTI TCR (Vα2) on their cell surface, whereas unaffected splenic tissue in the same mouse does. Blue represents the isotype control with red being the OTI Vα2 TCR. A representative example of three mice is shown in **f** and **g**.

The OT1 TCR (Vα2) recognizes ovalbumin (ova) residues 257–264 (SIINFEKL) in the context of H2κ^b^ (ref. 14). In the absence of ova, the CD4NA/RAG2^−/−^/OTI mouse line do not develop lymphoid tumours, but instead present after a longer latency with (OT1 TCR negative) gastrointestinal stromal tumours, hepatocellular carcinomas and sarcomas, even though OTI TCR-positive T cells exist in the periphery (Supplementary Fig. 6, Supplementary Table 4). In contrast, the RAG competent CD4NA/OT1 line do produce ALK^+^ lymphomas, with a peripheral rather than thymic presentation, and a mean survival of 181 days (versus 88 days for the CD4NA mice (*P*=0.0001, Student's *t*-test); Fig. 4a). Whether this delay in tumour development is a true difference or purely a result of belated tumour detection (the thymic CD4NA tumours induce laboured breathing, whereas peripheral tumours in CD4NA/OTI mice can be more difficult to detect owing to asymptomatic growth) is not clear. However, analysis of the DN3 population in CD4NA/OTI mice in comparison with CD4NA mice showed increased cellular proliferation in the former, which could potentially reduce time available for the acquisition of tumour-inducing secondary genetic events (possibly RAG-mediated) allowing thymic escape and transformation in the periphery (Supplementary Fig. 7). However, the CD4NA/OTI mice have apparently normal T-cell development profiles although total thymic cellularity is increased (Supplementary Fig. 8A–C). Notably, the peripheral tumours in the CD4NA/OTI line more closely replicate the histopathological presentation of the human disease (Table 2, Fig. 4b). All the tumours are positive for ALK staining by immunohistochemistry and histological analysis showed cells with eosinophilic cytoplasm and reniform nuclei, resembling the classic ‘hallmark' cells of human ALCL (Fig. 4b)^19^. Despite the presence of RAG in these mice, the tumours did not display endogenous TCRβ rearrangements, suggesting that they arose from the thymic populations expressing the OTI transgenic TCR, which is known to suppress endogenous rearrangements mediated by RAG (Fig. 4c). Expression of the OTI TCR (still in the absence of ova) would signal thymic emigration in the absence of other TCR rearrangements. However, the tumours rarely expressed the transgenic Vα2 TCR on the cell surface (only one out of 10 mice analysed; Fig. 4d, Table 2) even though Vα2 expressing T cells co-exist in unaffected organs of tumour-bearing mice (Fig. 4e) and are present in the periphery of pre-tumorigenic mice (Supplementary Fig. 6B), suggesting that its expression had been lost. Intracellular CD3 could be detected, but surface CD3 was not expressed on the tumours and there was an absence of intracellular Vα2 TCR even though OTI transcripts could be detected (Supplementary Fig. 8D,E). This ‘null' cell surface phenotype (Fig. 4d) is strikingly reminiscent of human ALK^+^ ALCL. It has been reported that the IL2R and molecules downstream of TCR-ligation induced signalling are silenced by epigenetic mechanisms in ALCL^10^,^20^, suggesting that TCR-induced signalling acts as a tumour-suppressive mechanism.

As the CD4NA/RAG2^−/−^/OTI transgenic mice do not develop lymphoid tumours, it was possible that RAG was acting as an essential tumour promoter in the NPM–ALK/OTI mouse. RAG1 transcripts are not produced by human ALCL tumours (Supplementary Fig. 3B) (even though RAG2 transcripts are detectable in our murine NA/OTI tumours, Supplementary Fig. 8E). RAG is normally lost with expression and cognate signalling of a functional TCR, so its absence in human ALCL is compatible with mature post-thymic cells but does not exclude RAG expression at an earlier stage of ALCL development. Alternatively, B-cells (which are not produced on the RAG^−/−^mouse background) might be an essential contributor to tumour development although there was no significant B-cell infiltration in tumours arising in the CD4NA/OTI mice (Supplementary Fig. 8F).

To assess the role of TCR–Ag interaction in peripheral ALCL development, we exposed NA/OT1 RAG competent mice to MHV-OVA (MHV68; ref. 21). Strikingly, this abrogated ALCL development in favour of hepatocellular carcinomas and sarcoma, as seen in the NA/RAG2^−/−^/OT1 mice (Fig. 4f, Supplementary Table 4). This demonstrates that cognate peripheral TCR signalling in NPM–ALK expressing cells is not compatible with ALCL development and/or survival. As expected, the OTI Vα2 TCR was not expressed on the surface of tumours developing in these mice, whereas it was present in unaffected haematological tissue (Fig. 4g).

Taken together, these data demonstrate that NPM–ALK permits bypass of thymic β-selection, but that TCR signalling is required for thymic egress and development of peripheral ALCL, the latter of which is dependent on subsequent abrogation of the TCR (Supplementary Fig. 9). Indeed, the analysis of 16 human ALK^+^ALCL by double staining for CD30 and the TCRβ chain showed an absence of tumour cells co-expressing both proteins, again suggesting that the presence of a TCR is not compatible with tumour cell survival in the presence of NPM–ALK (Supplementary Fig. 3A).

## Discussion

It has been notoriously difficult to recreate the clinical and immunophenotypic features of human ALCL in transgenic mouse models^12^,^22^,^23^,^24^,^25^. We now demonstrate that CD4-driven NPM–ALK can lead to both bypass of the RAG2^−/−^-mediated murine DN3 thymic maturation block and to the development of cortical thymic lymphoma. However, TCR expression is required for thymic egress and development of peripheral tumours which, for the first time, histologically resemble human ALK^+^ ALCL. The fact that these peripheral murine tumours cannot develop in the presence of ova-induced stimulation of the transgenic TCR and have lost endogenous TCR expression is strikingly similar to the widely recognized absence of TCR expression and signalling^9^,^10^, despite prior clonal TCR rearrangement, in human ALCL, and suggest that persistent co-existence of TCR and NPM–ALK-driven signalling in the periphery is not compatible with ALCL development and/or survival. Immunogenetic analysis of human ALCL demonstrated that the majority have major in-frame, potentially selected, TCRα receptor rearrangements in the absence of comparable TCRβ rearrangements, in keeping with the absence of detectable TCRβ protein, compatible with replacement of TCRβ by NPM–ALK in the development of classical ALCL.

NPM–ALK allowed cells to undergo asynchronous differentiation through thymic β selection in the absence of TCRβ rearrangement in the RAG2^−/−^ model, with expression of Notch1 (unlike rag2^−/−^ mice) and its downstream CD98 and CD71 nutrient transporters^26^ but maintenance of CD44 and CD117 expression, in keeping with previous identification of an ETP transcriptional signature in ALCL^11^. This indicates that the initial stages of ALCL development occur in the thymus and is compatible with the identification of a mediastinal mass in 50% of pediatric ALCL and high-level expression of Notch1 in ALCL^18^,^27^. As such, NPM–ALK signalling must be able to at least partially mimic pre-TCR signalling and allow maturation to TCRα rearrangement, including in the apparent absence of major clonal TCRβ rearrangement, as identified in 67% of (TCRαβ and TCRγ-only) human ALCL. NPM–ALK does not appear to prevent on-going TCRβ rearrangement in human ALCL, as many cases demonstrate clonal populations by PCR (but not by high-resolution CGH) and as such are probably present in 1–20% of the DNA. It is not possible to determine whether these minor rearrangements originate within the ALCL population or represent tumour infiltrating reactive T lymphocytes, but we favour the former as tumour infiltrating reactive T lymphocytes should be detectable with TCRαβ-specific antibodies by immunohistochemistry, which was not the case.

The delayed onset of lymphoma development in the NA/RAG2^−/−^ model and its absence in the NA/RAG2^−/−^/OT1 model would suggest that TCR signalling (or other RAG induced recombination) contributes to lymphomagenesis, although whether this is due to other, off-target RAG effects remains to be investigated. RAG activity in T and B lymphoid precursors carries the risk of creating recombinase-mediated abnormalities that might contribute to lymphomagenesis, as recognized in acute lymphoblastic leukemia^28^. An alternative explanation might lie in the ability of RAG proteins to induce DNA strand breakage at (cryptic) recombination signal sequences, which can lead to the production of oncogenic chromosomal translocations. It is unlikely that the absence of B lymphocytes is the explanation for failure to develop lymphoma in the absence of RAG, as there was no significant B-cell infiltration in tumours arising in CD4NA/OTI mice.

In the present model, RAG is dispensable for thymic tumours arrested before positive and negative selection in DP cortical thymocytes, but is required for peripheral tumours, as evidenced by the absence of lymphoma in NA/RAG^−/−^/OT1 mice, in keeping with a role for recombinase-mediated events in ALCL development. The fact that development of peripheral ALCL in the recombinase competent NA/OT1 mice was associated with loss of TCR transgene expression, and that induction of constitutive TCR signalling by exposure of NA/OT1 mice to ova abrogated peripheral ALCL development, suggest that TCR-induced signalling acts as a tumour-suppressive mechanism in the presence of NPM–ALK in peripheral T cells. ‘Immune-surveillance' or tumour suppressor function in ALCL has been ascribed to IL2Rγ signalling and NPM–ALK downregulates TCR-related signalling molecules by kinase-dependent, STAT3-mediated, gene transcription and/or epigenetic silencing^10^,^20^.

One possible explanation is that TCR^+^ ALK^+^ cells can leave the thymus and survive in the periphery, but that TCR signalling triggered by antigen stimulation leads to clonal deletion in the presence of oncogenic ALK. Abrogation of TCR signalling, as seen in human ALCL, and re-created in the RAG-competent NA/OT1 ‘silent' transgene in the absence of ova, would leave ALK signalling unopposed, thus favouring lymphoproliferation. It is possible that Ag (or other) T-lymphoid stimulation within a certain context, in addition to inducing expression of ALCL-hallmark activation markers such as CD30, perforin and Granzyme B (ref. 3), and initial TCR signalling, may in some way subsequently trigger TCR silencing and unopposed ALK signalling. In keeping with this, an antigenic stimulus triggering a strong immune response before ALCL onset, such as systemic ALK-positive ALCL skin lesions occurring after an insect bite, has been described^29^.

NPM–ALK can also lead to ALCL development in T-lymphoid subsets other than TCRαβ precursors, as 19% of human cases expressed a uniform TCRγδ immunogenetic profile, with thymic type Vδ1-Jδ1 rearrangements^30^. Naive TCRγδ cells produce IL-17, and a TH17 profile has been identified by transcriptional profiling in ALCL^31^. Eight ALCL (14%) expressed combinations of CD2, CD4, CD5 or CD7 but no detectable TCR rearrangements and as such correspond to the immunogenetic equivalent of null ALCL, which could result from transformation of NK or other innate immune subsets. These cells respond to an inflammatory cytokine environment rather than being classically TCR/MHC-restricted. However, the majority (80%) of human ALCL do demonstrate immunogenetic evidence of at least transient TCR expression and selection during ALCL development.

In normal TCRαβ lineage thymocytes, absent or excessive TCR signalling leads to cell death, as part of positive and negative selection of the TCRαβ repertoire. Our data suggest that ALK-driven signalling in the absence of RAG can replace an absent thymic pre-TCR proliferative signal, with uncontrolled cortical thymocyte expansion (as seen in the NA/RAG^−/−^ model) as there is no mechanism for TCR-mediated negative selection. Co-existence with a functional TCR allows thymic egress but subsequent cell death in the presence of simultaneous TCR and NPM–ALK signalling, by a process analogous to thymic negative selection. As such, the TCR acts as a tumour suppressor, which must be downregulated for development of classical, peripheral ALCL. These observations clearly need further experimental investigation of the precise mechanisms underlying peripheral counter-selection but the current data describe a murine model, which for the first time closely resemble human ALCL and which will be useful for evaluation of novel therapeutic agents.

Furthermore, this work raises the possibility that children affected by ALCL harbour ‘lymphoma-initiating cells' in the thymocyte population that could contribute to relapse after chemotherapy has apparently cleared the disease. Previously, we have shown that ALCL-propagating cells express a gene signature associated with an ETP in keeping with a presumed thymic origin as described in the current model^32^. Indeed, NPM–ALK transcripts have been detected in 2% of newborn cord blood^33^ and expression of NPM–ALK is regulated by the endogenous *NPM1* promoter, which drives ubiquitous expression of NPM1 including in thymic progenitors (Supplementary Fig. 10). Thus, it is not unreasonable to speculate that NPM–ALK is likewise expressed in this cellular population.

## Methods

### Human ALCL biopsies

Tumour biopsies were obtained with informed consent at diagnosis from 57 ALK-positive ALCL centralized in the Department of Pathology Universitaire du Cancer in Toulouse in accordance with institutional review board-approved protocols (DC 2009-989) and in accordance with the Helsinki Declaration of 1975, as revised in 2000. All but two (one soft tissue mass, one colon tumour) were lymph node biopsies. Among the 57 patients, 35 were male and 22 female. The study group included 48 paediatric ALCL (less than 18 years old) and 14 adult ALCL. The diagnosis of ALCL was made on formalin-fixed paraffin-embedded tissues and based on morphologic and immunophenotypic criteria, as described in the last WHO classification^34^, using a large panel of monoclonal antibodies against CD30/BerH2, ALK, EMA, several T cell (CD2, CD3ɛ, CD4, CD5, CD7, CD8, CD43) and B-cell markers (CD20, CD79a) and cytotoxic molecules (Perforin, Granzyme B, TiA1). Cases were classified as T/NK lineage if they reacted with one or more antibodies against the T- or NK-cell antigens CD2, CD3ɛ, CD4, CD5, CD7, CD8, CD43 or cytotoxic molecules and lacked reactivity for the CD20 and CD79a B-cell-associated antigens. A null phenotype was assigned to cases that did not express either T/NK- or B-cell-associated markers. All the cases were reviewed by an international pathology panel. Fifty cases expressed NPM–ALK protein, four TPM3–ALK and two ATIC–ALK; in one case, the ALK partner was not identified.

Frozen tumour samples with informed consent were retrieved from CHU de Toulouse tumour tissue bank, in accordance with institutional review board-approved protocols (DC 2009-989) and in accordance with the Helsinki Declaration of 1975, as revised in 2000. The percentage of malignant cells was assessed on frozen sections by ALK1 or CD30 staining and was greater than 40% for all the selected cases. Total DNA extraction was performed from 10 frozen sections of tumour biopsies (10 μm thickness each), using the QIAamp DNA Mini kit (Qiagen, Courtaboeuf, France), following the manufacturer's protocol. Total RNA extraction was performed from 40 frozen sections of tumour biopsies (5 μm thickness each), using Trizol total RNA isolation reagent (Invitrogen, San Diego, USA) following the manufacturer's protocol. The concentration was quantified by NanoDrop Spectrophotometer (NanoDrop Technologies, Wilmington, USA). RNA integrity was assessed on an Agilent 2100 Bioanalyzer (Agilent, Massy, France). DNA integrity was assessed by 6% polyacrylamide gel electrophoresis. Quantification of RAG1 transcripts was performed as previously described^35^.

Human thymi were obtained as surgical tissue discards from children, with informed consent from the parents and the ethical review board of Necker Enfants Malades Hospital at Paris Descartes.

### Genescan analysis of human TCR rearrangements

The TCRγ multiplex PCR were performed as described^36^. Briefly, 500 ng DNA was amplified for 35 cycles with 0.25 μM each primer, 2.5 mM MgCl_2_ and 1 U Taq Gold (Life Technology).

The TCRβ and TCRδ multiplex PCRs were developed within the Biomed-2 BMH4-CT98–3936 Concerted Action @JJM^37^. Briefly, 100 ng DNA was amplified for 35 cycles in the presence of 0.2 μM each primer, 2 mM MgCl_2_ and 1 U Taq Gold (Perkin Elmer). TCRβ gene configuration was assessed with a three-tube multiplex PCR, two of which contained 27 Vβ family-specific upstream primers with either nine (PCR A) or four (PCR B) downstream Jβ primers (Supplementary Table 5). The third (PCR C) contained all 13 Jβ primers and Dβ1 and Dβ2 upstream primers (Supplementary Table 5). TCRβ and TCRδ PCR products were analysed by heteroduplex analysis and Genscan analysis of multiplex fluorescent PCR by separation of fluorochrome-labelled single strand (denatured) PCR products in a capillary sequencing polymer and detection via automated laser scanning. This results in a Gaussian distribution of multiple peaks representing many different PCR products in the presence of polyclonal rearrangements, but a discrete, narrow peak if a clonal population represents at least 1–5% of the DNA.

The large number of Vα and Jα segments precludes PCR detection of clonal rearrangements from DNA, although TCRα VJC transcripts can be detected from cDNA. TCRα rearrangements were amplified from cDNA using Cα and Vα primers in five multiplex RT–PCR reactions^38^. RNA was isolated using an RNeasy kit according to the manufacturer's instructions (Qiagen, Courtaboeuf, France) and 1 μg of total RNA was converted into cDNA using SuperScript III RT (Invitrogen, San Diego, USA). RNA quality and quantity was assessed relative to the *ABL* housekeeping gene, according to protocols developed within the Europe against Cancer network^39^ on an AB 7900HT (Applied Biosystems, Foster City, CA, USA). RNA (20 ng) was amplified in each PCR with 1 U of Hotstart Taq (Qiagen, Courtaboeuf, France), 10 mM dNTPs, 2 mM of MgCl_2_, 20% Q solution, and 10 pmol of each primer. The Taq polymerase was activated for 15 min at 95 °C, followed by 37 cycles of 94 °C for 30 s, 63 °C for 45 s and 72 °C for 1.5 min. The final elongation step was 72 °C for 10 min. The negative control consisted of RNA isolated from peripheral blood mononuclear cells from donors and the positive control was RNA isolated from the Jurkat cell line. Primers sequences used for the TCRA transcript amplification are listed in Table 3.

### CGH analysis of TCR rearrangements

DNA at diagnosis were analysed by array-comparative genomic hybridization (array-CGH, Agilent human CGH MicroArray, 180K) and by High Density Affymetrics Cytoscan (2.7 million probes) according to the manufacturer's recommendations. Deletion of TCR loci, as a reflection of V(D)J recombination, were defined by the CGH log_2_ copy number ratio, with deletion/rearrangement on one allele defined as 0.5 to 1.5, whereas log_2_ copy number ratios <1.5 were defined as biallelic deletions/rearrangements. Data are accessible on the NCBI GEO database under GSE67131.

### Transgenic mice

The CD4/NPM-ALK (CD4NA) mice were generated as previously described (kindly provided by Professor G. Inghirami, University of Turin, Italy)^12^. NPM–ALK/OTI (CD4NA/OTI) mice were generated by crossing the CD4NA line to the OT1 transgenic T cell line (kindly provided by Professor G. Griffiths, University of Cambridge, UK)^14^. NPM–ALK/RAG2^−/−^ (NARAG2^−/−^) mice were generated by crossing the CD4NA line to the RAG2^−/−^ line (kindly provided by Professor C. Rudd, University of Cambridge, UK). NPM–ALK/RAG2^−/−^/OT1 (NA/OTI/RAG2^−/−^) mice were generated by crossing the NARAG2^−/−^ line with the OT1 transgenic T cell line that had been backcrossed to the RAG2^−/−^ line. OTINA mice were exposed to 2 × 10^4^ p.f.u. MHV-Ova (MHV68), intranasally on three occasions at monthly intervals starting at 6 weeks of age. Virus was prepared as described in ref. 21 and was provided by Dr P. G. Stevenson, University of Cambridge. All the mice were housed under specified pathogen free conditions in ventilator cages within a barrier facility at the University of Cambridge and were maintained on a C57/Bl6 genetic background and were analysed at 4–12 weeks of age unless indicated. All the procedures were conducted under UK Home Office license 80/2630 at the University of Cambridge according to UKCCR guidelines.

Genotyping of the mice was performed by PCR on DNA extracted from ear biopsies, and the PCR products were separated by agarose gel electrophoresis as described previously^12^,^40^,^41^ using the following primers: OT1 FWD 5′- ACGTGTATTCCCATCTCTGG -3′, OTI R 5′- CTGTTCATAATTGGCCCGA -3′; NPM–ALK FWD 5′- TCCCTTGGGGGCTTTGAAATA -3′, NPM–ALK RVS 5′- CGAGGTGCGGAGCTTGCTCAG -3′; RAG A 5′- GGGAGGACACTCACTTGCCAGTA -3′, RAG B 5′- AGTCAGGAGTCTCCATCTCACTGA -3′ and NeoA 5′- CGGCCGGAGAACCTGCGTGCAA -3′.

### Histology

Mice displaying clinical signs were euthanized and post-mortem performed examining all tissues for tumour formation. Tumours and/or associated tissues were fixed in 10% formalin, and subsequently paraffin embedded, sectioned and stained with haematoxylin and eosin for histological examination. All tissue processing was carried out at either the Department of Pathology, University of Cambridge, UK, or the Medical University of Vienna, Austria and sections were examined independently by at least two histopathologists.

### Immunohistochemistry

Immunohistochemistry was carried out with the following antibodies: CD30 (Monoclonal Mouse Anti-Human; Clone Ber-H2, 1:50, Dako, Denmark); TCR β-F1 (Monoclonal Mouse Anti-Human, Clone 8A3, 1:100, Thermo Scientific, IL 61105 USA); ALK1 mAb (1:100 kindly provided by Dr Karen Pulford, University of Oxford) as described in ref. 42.

### Cell lines

The human cell lines SUDHL-1, Karpas-299, HPB-ALL, DND41 and Jurkat were purchased from the DSMZ and were cultured in RPMI1640 supplemented with 10% FCS. The COST and PIO ALCL cell lines were developed in the Lamant laboratory and were grown in ISCOVE medium (Invitrogen, Cergy Pontoise, France) supplemented with 10% FCS. All the cell lines were subject to quarterly mycoplasma testing and are not on the ICLAC and NCBI Biosample misidentified cell list.

### Flow cytometry

Single-cell suspensions were obtained from isolated tissue samples. Cells were washed, counted and stained with the following murine phycoerythrin, fluorescein isothiocyanate, allophycocyanin or cyanine 7 (Cy7)—conjugated antibodies: CD4, CD8, CD44, CD25, CD30, CD117, CD3, Vα2 TCR, Notch1 (BD Biosciences, Oxford, UK). After staining (30 min at 4 °C, 1:1,000), cells were washed and analysed using fluorescence-activated cell sorter (FACS) Canto or Fortessa flow cytometers (BD Biosciences). For intracellular stain, the cells were fixed in 0.01% formaldehyde for 10 min, permeabilized in 0.5% Tween 20 v/v in PBS (15 min in the dark at RT), cells were washed in PBS 0.1% Triton and re-suspended in 100 μl of 0.1% Triton in PBS, cells were stained and analysed as previously described. Alternatively, cell populations were sorted using a FACS Aria machine as detailed in ref. 11.

### RT–PCR analysis

RNA was extracted from cells using an RNeasy Micro Kit (Qiagen) according to the manufacturer's instructions and converted to cDNA using Superscript II reverse-transcriptase (RT) (Invitrogen), with dNTPs and random hexamers. PCR was carried out using primers specific to NPM–ALK^24^, RAG2 or Vα2 (OTI transgene) (OT1 FWD 5′- ACGTGTATTCCCATCTCTGG -3′, OTI R 5′- CTGTTCATAATTGGCCCGA -3′; NPM–ALK FWD 5′- TCCCTTGGGGGCTTTGAAATA -3′, NPM–ALK RVS 5′- CGAGGTGCGGAGCTTGCTCAG -3′; RAG A 5′- GGGAGGACACTCACTTGCCAGTA -3′, RAG B 5′- AGTCAGGAGTCTCCATCTCACTGA -3′ and NeoA 5′- CGGCCGGAGAACCTGCGTGCAA -3′).

### Murine TCR rearrangement analysis

DNA was extracted from 5 × 10^6^ murine tumour cells or thymocytes using the DNeasy Blood and Tissue Kit (Qiagen). PCR was performed as previously described^13^,^43^. Briefly Vβ2 (5′- GTAGGCACCTGTGGGGAAGAAACT -3′) or Dβ2 forward (5′- GGGTCACTGATACGGAGCTG -3′) primers and Jβ2 reverse (5′- TGAGAGCTGTCTCCTACTATCGATT -3′) primer (for the V-J and D-J reactions, respectively) were used to amplify rearranged TCRβ regions, which were then separated by electrophoresis. Full uncropped images are shown in Supplementary Figs 11–14 for all PCR data presented.

### RQ-PCR of RAG status

RAG expression transcript Ct values were normalized for RNA quality and quantity relative to Abl as previously described^35^. One microgram of total RNA was reverse transcribed as described^39^ and RNA quality and quantity was assessed relative to the *ABL* housekeeping gene, on an ABI PRISM 7900HT (Applied Biosystems, Foster City, CA, USA). Primers used for the analysis of Rag1 were: RAG1 sense, 5′- AGCCTGCTGAGCAAGGTACC -3′; antisense, 5′- GAACTGAGTCCCAAGGTGGG -3′; probe, Fam-5′- AGCCAGCATGGCAGCCTCTTTCC -3′-Tamra

### Immunohistochemistry

Double-staining immunohistochemistry was performed using the automated Ventana Benchmark platform (Ventana Medical Systems Tuscon, AZ, USA) by sequential staining for both antibodies (first TCR β-F1, then CD30). Pre-treatment was carried out with enzyme I (Leica AR9551) for TCR β-F1 and Epitope Retrieval 1 (Leica AR9961) for CD30. Staining was developed using the Bond Polymer Refine detection kit (DAB for TCR β-F1) and Bond Polymer Refine Red detection kit (AP Fast Red for CD30) according to the manufacturer's recommendations. ALK1 staining was performed using a standard protocol. HIER procedure was performed for antigen retrieval using Target Retrieval Solution (DAKO S2369). Development was carried out using the IDetect Super Stain System HRP (ID Laboratories, IDSTM007). The signal was visualized with 3-amino-9-ethylcarbazole (ID Laboratories, BP1108).

### Irradiation and intravenous injection of thymocytes

Before injection, wild-type recipient mice were exposed to two rounds of irradiation to a total of 11 Grays to clear the host bone marrow. Thymocytes were obtained from the thymus of a CD4NA mouse that showed signs of dysplasia as assessed by cell surface protein expression. CD44^hi^ and CD44^lo^ populations were sorted into PBS using a FACS Aria (BD). Sorted thymocytes from CD45.2 donor BL/6 mice (3 × 10^5^) were then mixed with donor CD45.1 wild-type bone marrow cells (6.6 × 10^4^) and intravenously injected into irradiated CD45.2 recipients.

### Luciferase assay

In each transfection reaction 1 × 10^7^ Jurkat cells were mixed with plasmid DNA in a 0.4 cm electrode gap Gene Pulser cuvette (40 μg of pGL2-CD44-firefly luciferase, 2 μg of pRL-TK vector, and varying amounts of an NPM–ALK vector as indicated) and electroporation performed on the Gene Pulser II (Bio-Rad), at 0.25 kV and 950 μF for ∼25 ms. After 48 h in culture, firefly and renilla luciferase were exposed to their relevant substrates using a Stop and Glo kit (Promega), and the output measured on the Victor^3^ multi-label counter (Perkin Elmer, Waltham, MA) as described previously^16^.

### *In vivo* EdU analysis of cellular proliferation

Mice were injected intraperitoneally with 300 μl of a 10 mM EdU solution in PBS. After 3 h, mice were killed and the thymus of each mouse was isolated before FACS staining for T cell markers as described in the main text. After staining (30 min at 4 °C), cells were washed and then fixed, permeabilized and EdU incorporation detected using a Click-iT EdU Flow Cytometry Assay Kit (Invitrogen). The amount of EdU incorporated was then measured by Flow Cytometry.

### Statistics

Data were analysed using a two-tailed Student's *t*-test (assuming equal variance) or log-rank test, as indicated using GraphPad Prism 6 software (La Jolla, CA).

## Additional information

**Accession codes:** The aCGH data have been deposited in the NCBI GEO database under accession code GSE67131.

**How to cite this article:** Malcolm, T. *et al*. Anaplastic large cell lymphoma arises in thymocytes and requires transient TCR expression for thymic egress. *Nat. Commun.* 7:10087 doi: 10.1038/ncomms10087 (2016).

## Supplementary Material

Supplementary Information Supplementary Figures 1-14 and Supplementary Tables 1-5

## Figures and Tables

**Table 1:** Summary of ALK^+^ ALCL cases analysed.

**TCR-γδβ PCR classification**	**# ALK**^**+**^ **ALCL (% total cases)**	**CD2**	**CD3**	**CD5**	**CD7**	**CD4**	**CD8**	**CGH TCR α/β**	**CGH TCR β**	**TCR Vα-Cα transcripts**
TCRαβ (γ^VJ^δ^−^β^VDJ^)	32 (56%)	36% 25	23% 30	34% 29	50% 20	44% 27	48% 27	16/17 Vα-Jα	3/17	10/15
TCRγ only (γ^VJ^δ^−^β^−/DJ^)	6 (11%)	60% 5	0% 8	50% 6	33% 3	50% 6	20% 5	2/4 Vα-Jα	0/4	5/6
TCRγδ (γ^VJ^δ^VDJ^β^DJ/VDJ^)	11 (19%)	50% 8	40% 10	30% 10	67% 6	57% 7	0% 6	4 VJ/DJδ, 1 DJδ, 1 GL/6	0/6	0/1
TCR GL (γ^−^δ^−^β^−/DJ^)	8 (14%)	50% 6	0% 6	29% 7	33% 6	50% 8	14% 7	0/4	0/4	ND
Total ALCL	57 (100%)	77% 44	20% 54	35% 52	38% 45	48% 48	33% 45	31 cases tested	31 cases tested	22 cases tested

Classification of TCR status as determined by genescan PCR analysis of γβδ genes. To refine the TCRα/δ and the TCRβ status 180K Agilent CGH array analysis was performed for 31 ALK^+^ ALCL and RT–PCR detection of clonal TCRα transcripts from cDNA using Vα and Cα primers, was performed for 22 cases with available RNA, ND, not determined. Immunohistological expression of T-lymphoid markers is given as a percentage of the number of cases analysed (shown below percentage).

**Table 2:** Location and phenotype of mouse tumours isolated from mice of the indicated genetic backgrounds.

**Mouse**	**NPM**–**ALK**		**NPM**–**ALK/RAG2**^**−/−**^		**NPM**–**ALK/OTI**	
	**Location**	**Phenotype**	**Location**	**Phenotype**	**Location**	**Phenotype**
1	Thymus	4,8,44,25,117,30 (DP)	Thymus	4,8,44 (DP)	LN (c,a,i,m), Spleen	4,44,25,117 (SP)
2	Thymus	4,8,44,25,117 (DP)	Thymus	4,44,25,117 (SP)	Thymus	4,44,117 (SP)
3	Thymus	4,8,117 (DP)	Thymus	4,8,44,25 (DP)	LN (c,a,i,m), Spleen	Null,25
4	Thymus	4,8,44,25,117,30 (DP)	Thymus	4,8,44,25 (DP)	LN (m), Spleen	Null,44
5	Thymus	4,44,117,30 (SP)	Thymus	4,8,44 (DP)	LN (i,m), Spleen	Null,44,25,117
6	Thymus	44,25,117 (DN)	Thymus	4,44,25 (SP)	LN (c,i,md)	Null,44,25
7	LN (c,a,i,m), Spleen	Null	Thymus	4,8,25,30 (DP)	LN (c,i,m,md), Spleen	Null,44,25
8	Thymus	4,44,117 (SP)	Thymus	4,8,44,25,30 (DP)	LN (m), Spleen	4,44,Vα2 (SP)
9	Thymus	4,8,44,117 (DP)	Thymus	4,8,25 (DP)	LN (c,a,i,m)	Null,25
10	Thymus	4,44,25,117,30 (SP)	Thymus	4,8,44,25 (DP)	LN (c,a,i,m)	8,25,117 SP

a, axillary; c, cervical; CD, cluster of differentiation; DP, double positive; I, inguinal; LN, lymph node; m, mesenteric; md, mediastinal; SP, single positive.

A phenotype was assigned based on the cell surface expression profile with null relating to those tumours that were negative for all lineage defining markers (CDs 3, 4, 8 and B220). A representative set of 10 tumours for each genotype are shown. Where a cell surface protein is not mentioned in the table, it was not expressed on the tumour cells (of CDs 4, 8, 25, 44, 117, 30 and Vα2).

**Table 3:** Primer combinations used for multiplex analysis of human TCRα rearrangements.

***TCRA*** **PCR**				
Tube 1	Tube 2	Tube 3	Tube 4	Tube 5
Cα	Cα	Cα	Cα	Cα
Vα1-1	Vα3	Vα5	Vα2	Vα1-2
Vα8-4	Vα4	Vα8-1	Vα6	Vα8-2
Vα8-7	Vα9-2	Vα22	Vα7	Vα9-1
Vα12-1	Vα13-1	Vα24	Vα8-3	Vα10
Vα12-2	Vα16	Vα26-2	Vα8-5 (p)	Vα11 (p)
Vα12-3	Vα17	Vα27	Vα8-6	Vα13-2
Vα30	Vα18	Vα29	Vα19	Vα14
Vα35	Vα31 (p)	Vα32 (p)	Vα20	Vα15 (p)
Vα39	Vα33 (p)	Vα36	Vα23	Vα21
Vα40	Vα38-2	Vα37 (p)	Vα26-1	Vα25
Vα41		Vα38-1	Vα28 (p)	Vα34
