# Supplementary material for: Anaplastic large cell lymphoma arises in thymocytes and requires transient TCR expression for thymic egress
Source: Nat Commun. 2016 Jan 12;7:10087. doi: 10.1038/ncomms10087 (PMC4729925; doi:10.1038/ncomms10087)
Supplement: Supplementary Information — Supplementary Figures 1-14 and Supplementary Tables 1-5 [file ncomms10087-s1.pdf]

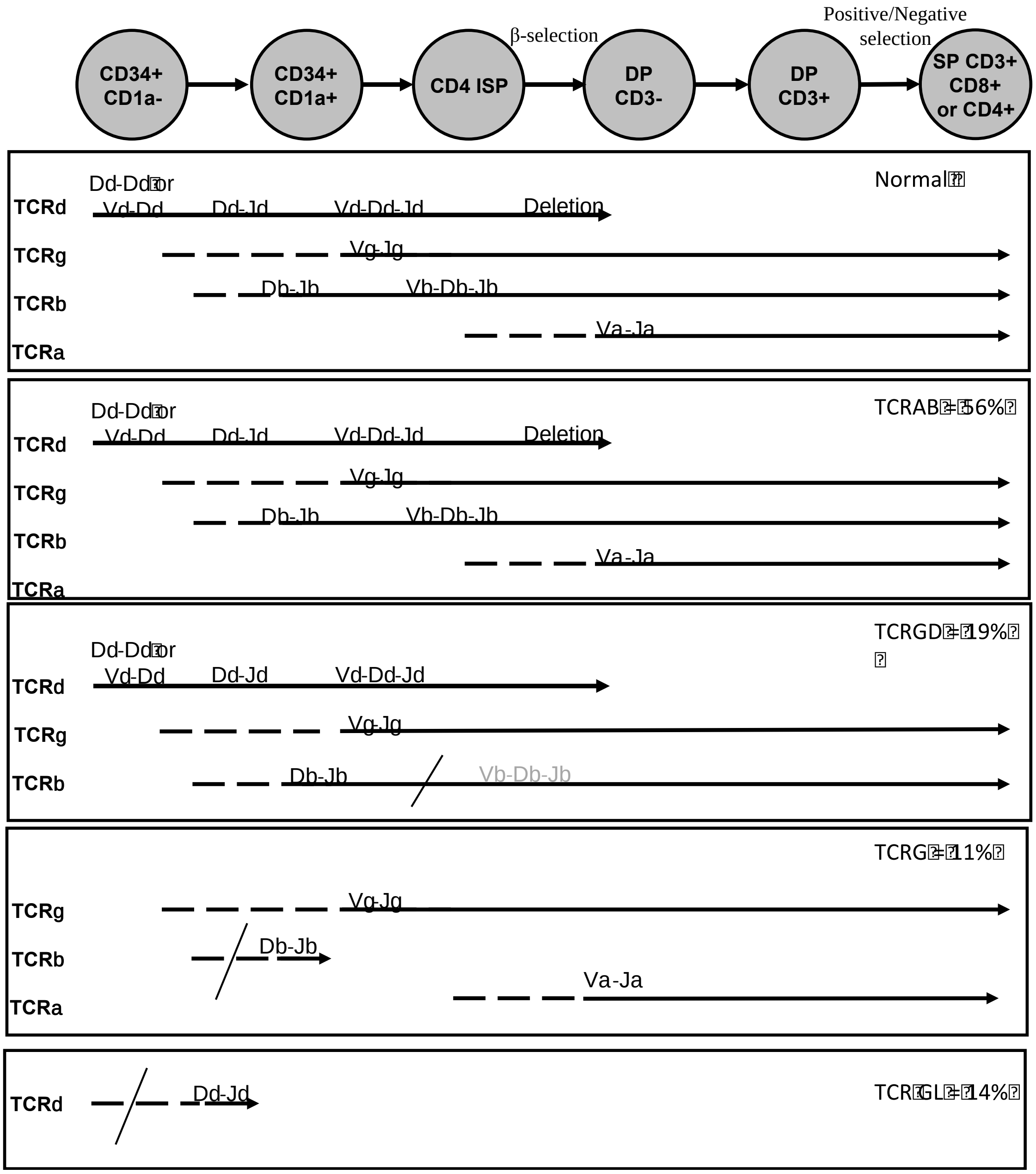

Supplementary Figure 1: Schematic summarising the categories of TCR rearrangement in human ALCL. During normal T cell development, rearrangement of TCR genes occurs in a temporal manner whereby rearrangement of δ precedes γ, which is followed by β and then α, the latter of which coincides with deletion of the δ locus. We classified the human ALCL tumours into 4 distinct immunogenetic profiles with regards to the presence or absence of TCR rearrangements. The TCRαβ category having a normal set of rearrangements consistent with an αβ T cell, TCRγδ displaying rearrangements of TCRδ and γ, but lacking TCRα rearrangements and having partial or full TCRβ, TCRγ having a full complement of rearrangements including deletion of the TCRδ locus but having partial or no TCRβ rearrangement (an alternative explanation is that this category lack TCRδ, β and α rearrangements with only TCRγ) and the GL category in which all TCR loci are germline with at most some partial TCRβ rearrangement detected in some cases.

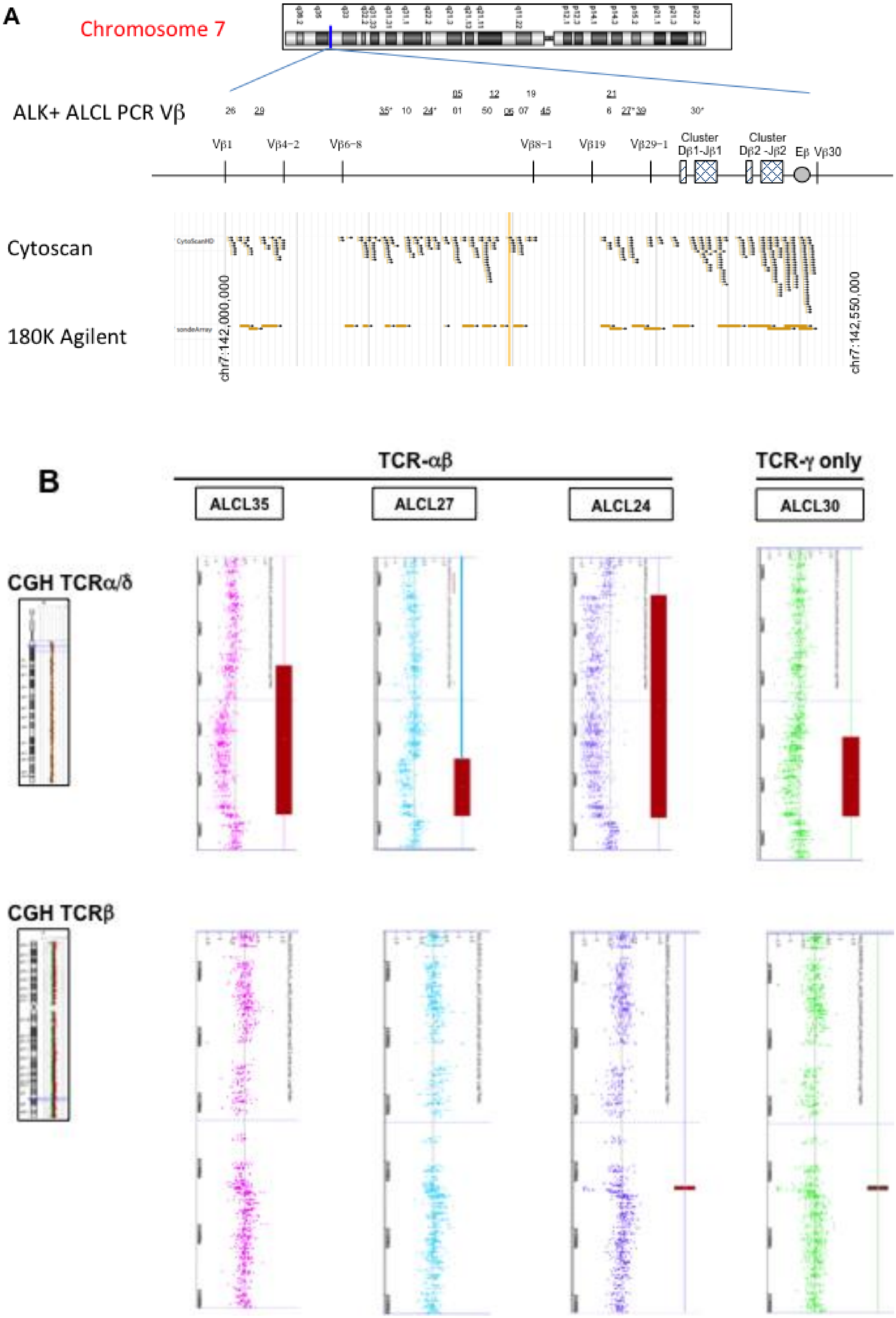

A
Chromosome 7
ALK+ ALCL PCR Vβ
Vβ1
Vβ4-2
Vβ6-8
Vβ8-1
Vβ19
Vβ29-1
Cluster Dβ1-Jβ1
Cluster Dβ2 -Jβ2
Eβ
Vβ30
Cytoscan
180K Agilent
chr7:142,000,000
chr7:142,550,000
B
TCR-αβ
TCR-γ only
ALCL35
ALCL27
ALCL24
ALCL30
CGH TCRα/δ
CGH TCRβ

Supplementary Figure 2: High density CGH array analysis of TCR rearrangements. (A) The chromosome coordinates of the TCRβ locus extremities are shown. Localisation of TCRβ rearrangements detected by PCR in ALK$^+$ ALCL relative to the resolution of Cytoscan High Density (upper panel) and 180K Agilent low density (lower panel) CGH probes on chromosome 7. The chromosome coordinates of the TCRβ locus extremities are shown. *ALCL analysed by both 180K Agilent and Cytoscan CGH; ALCL which were TCRβ VDJ positive by PCR but negative by 180K CGH are underlined. (B) High density CGH array of the TCRα and TCRβ loci. Vertical red lines correspond to TCRα Vα-Jα or TCRβ DJ rearrangements/deletions

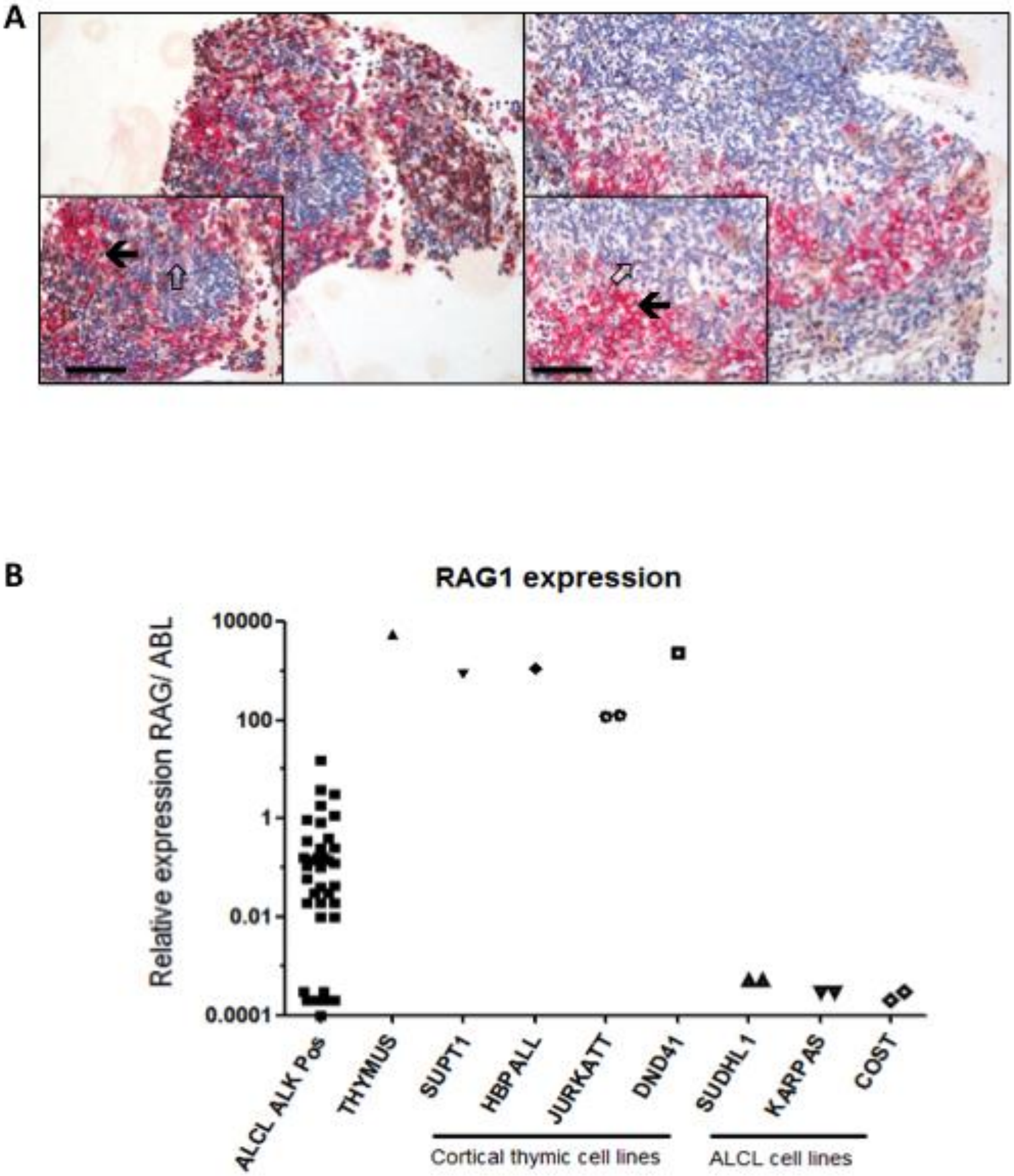

Supplementary Figure 3: ALK$^+$ ALCLs do not express the TCRβ chain or RAG1 transcripts. (A) Double staining for CD30 (red) and βF1 (TCRβ; brown) of 2 representative ALCL, ALK$^+$ showing variable, loosely to densely distributed CD30 positive tumour cells (filled arrows); intermingled with few to moderate numbers of βF1 positive lymphocytes (empty arrow). There are no CD30/βF1 co-expressing tumour cells. Magnification 200x and 400x (insert). These data are representative of 16 cases analysed. Scale bars represent 50 µm. (B) Transcript levels of RAG1 were determined by RQ-PCR in 52 ALCL tumours and immature, cortical thymic T-ALL or ALK$^+$ ALCL cell lines (data represent the means of duplicates) and expressed as ΔΔCt relative to ABL transcripts. This experiment was conducted on one occasion.

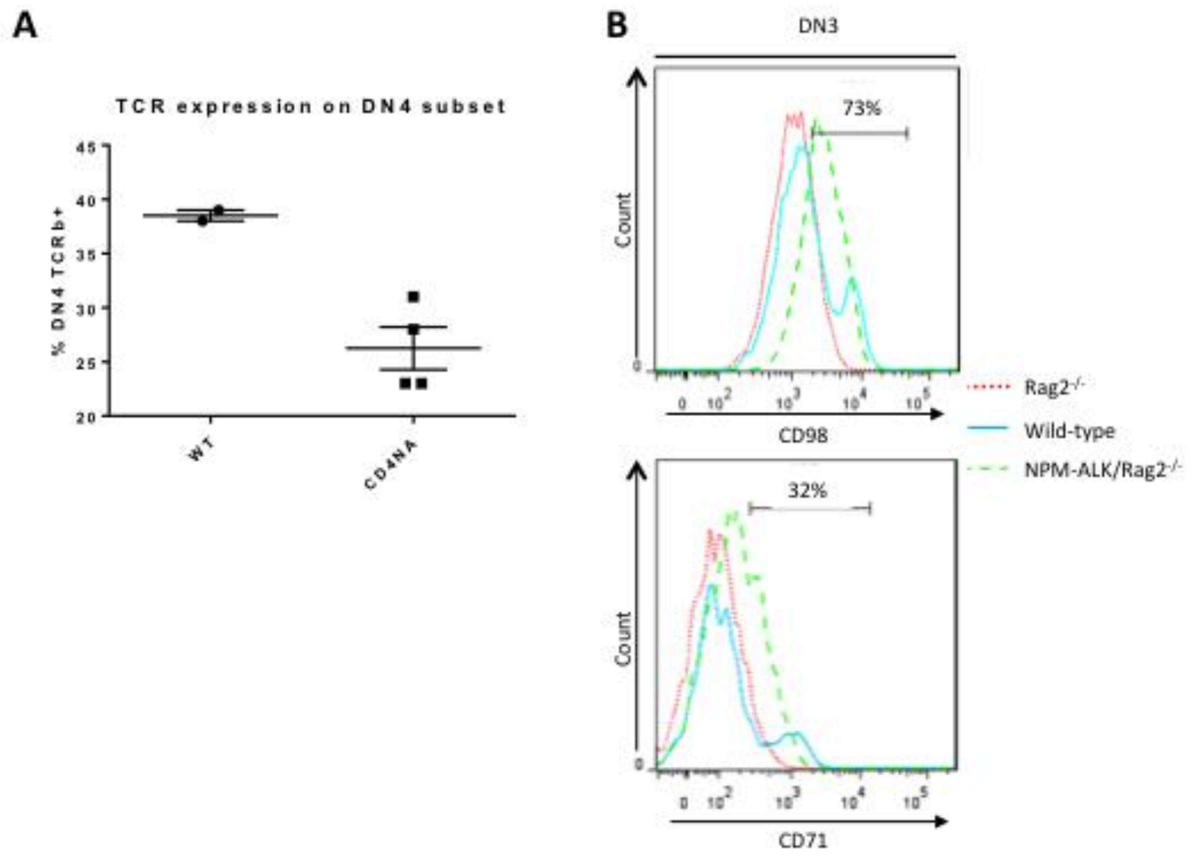

Supplementary Figure 4: A lower percentage of DN4 cells express TCRβ in the presence of NPM-ALK and DN3 thymocytes in CD4NA/RAG2$^{-/-}$ mice up-regulate expression of the nutrient transporters CD98 and CD71. (A) DN4 cells isolated from CD4NA and wild-type (WT) littermate control mice were analysed for expression of extracellular TCRβ, n=3 for each genotype and the experiment was repeated twice with these data being representative of 1 experiment, *p*=0.0147, Student’s t-test. Data represent the mean (horizontal bars) with the whiskers being the standard error of the mean. (B) A higher percentage of DN3 cells express CD71 and CD98 in NPM-ALK/RAG2$^{-/-}$ mice. Data are representative of 3 mice analysed of each genotype and gates are drawn according to isotype controls.

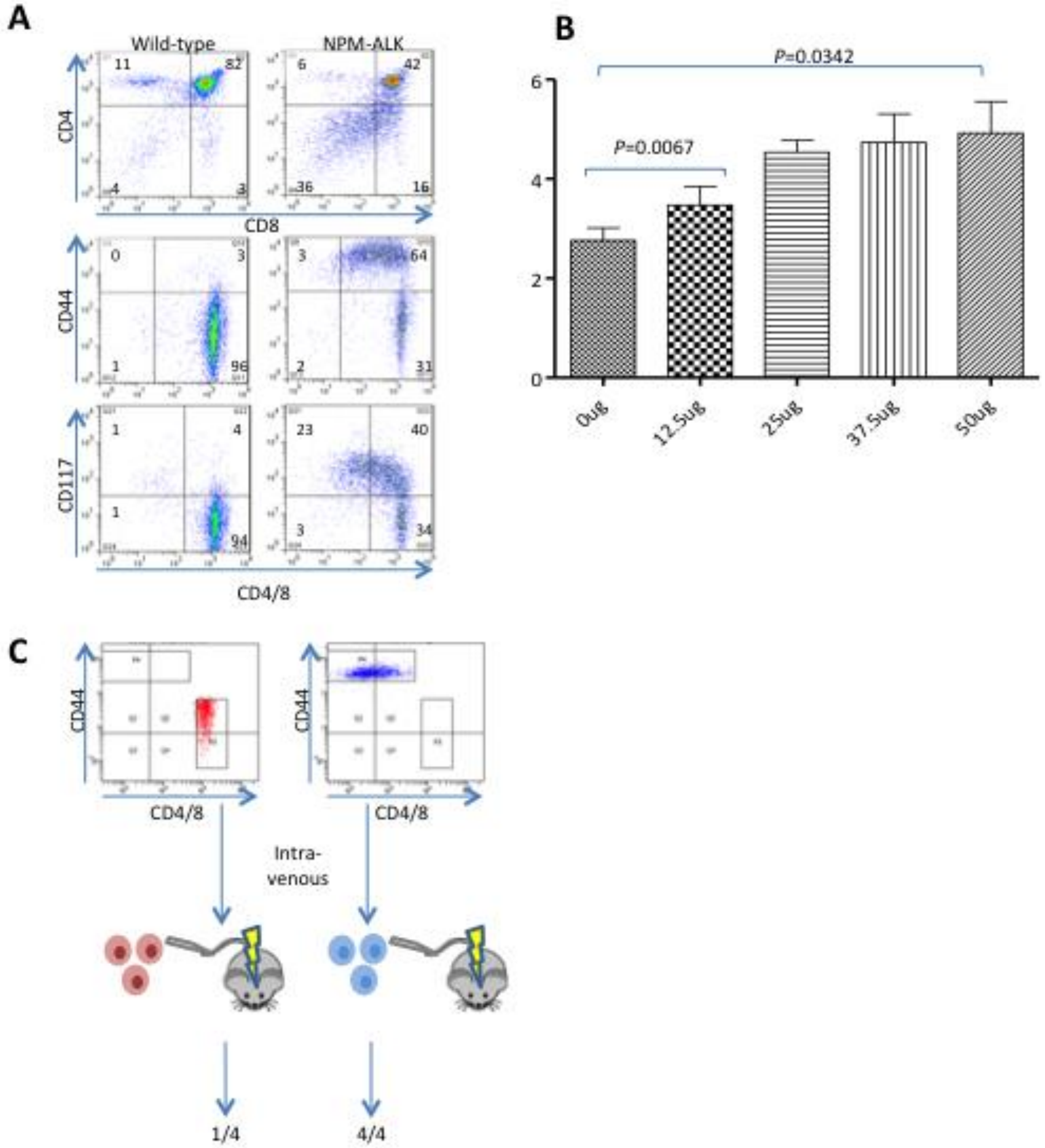

Supplementary Figure 5: An aberrant population of CD44^{hi} expressing thymocytes from CD4NA mice initiate tumour growth *in vivo*. (A) By 10 weeks of age an aberrant population of thymocytes emerges whereby CD44 and CD117 expression are retained on mature thymocytes. Numbers in the graph represent the % of total live thymocytes. Data are representative of at least 7 mice. (B) Jurkat T cells were transfected with a CD44-luciferase reporter construct (40µg), a pRL-TK vector (2µg) and NPM-ALK at the indicated concentrations and luciferase activity measured 48 hours later (firefly divided by renilla). Data are representative of those seen in at least three independent experiments and represent the standard error of the mean of triplicate counts. *P*-values were determined using a Students t-test assuming equal variance. Data represent the mean and (C) The CD44^{hi} population gives rise to tumours following intravenous injection of $3x10^5$ cells into each of 4 irradiated recipient mice. This experiment was repeated once with the same result.

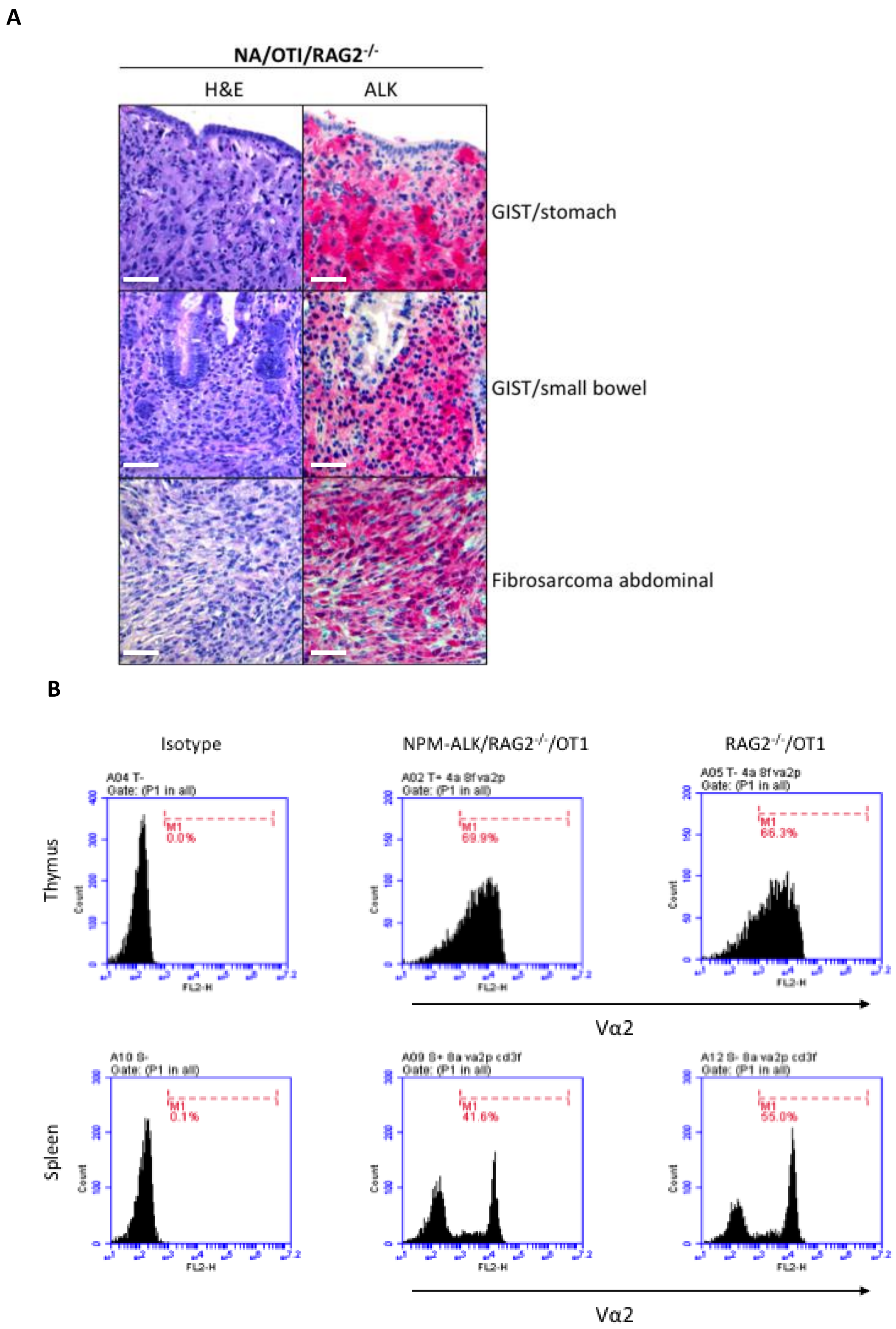

Supplementary Figure 6: CD4NA/RAG2$^{-/-}$/OTI mice develop sarcomas, gastrointestinal stromal tumours and hepatocellular carcinomas. (A) Representative H&E images and their ALK-stained counterparts of the sarcomas and gastrointestinal stromal tumours presenting in the CD4NA/OTI/RAG2$^{-/-}$ mice. Scale bars represent 50 µm (B) Vα2 expressing cells are detectable in the thymi and spleen of NPM-ALK/RAG2$^{-/-}$/OTI transgenic mice. A representative example is shown of at least 5 animals analysed.

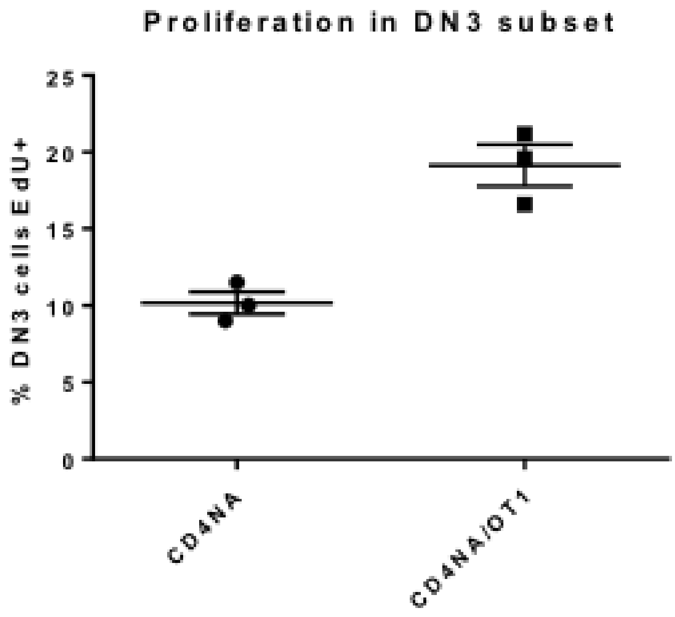

Supplementary Figure 7: NPM-ALK^{+} DN3 cells proliferate faster in the presence of the OTI transgene. DN3 cells were gated according to expression of CD44 and CD25 (CD3/4/8^{-} CD25^{+}CD44^{-}) and analysed for incorporation of EdU (y-axis), n=3 mice per group, *p*=0.0042, Student's t-test. Data represent the mean (horizontal bars) and standard error of the mean (whiskers).

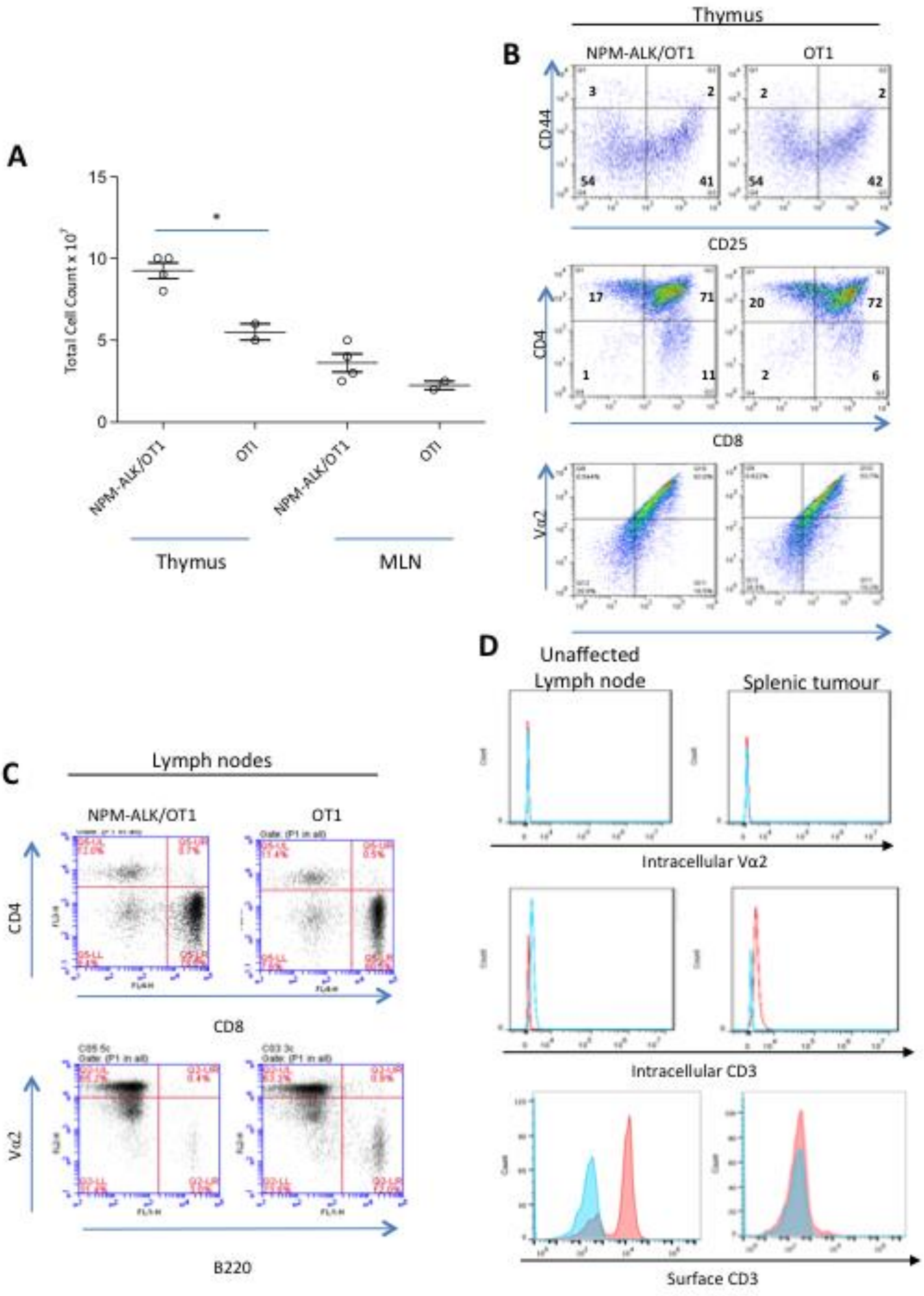
A
15
10
5
0
Total Cell Count x 10^7
*
NPM-ALK/OT1
OTI
NPM-ALK/OT1
OTI
Thymus
MLN
B
Thymus
NPM-ALK/OT1
OT1
CD44
3
2
54
41
2
2
54
42
CD25
CD4
17
71
1
11
20
72
2
6
CD8
Vα2
C
Lymph nodes
NPM-ALK/OT1
OT1
CD4
CD8
Vα2
B220
D
Unaffected
Lymph node
Splenic tumour
Intracellular Vα2
Intracellular CD3
Surface CD3

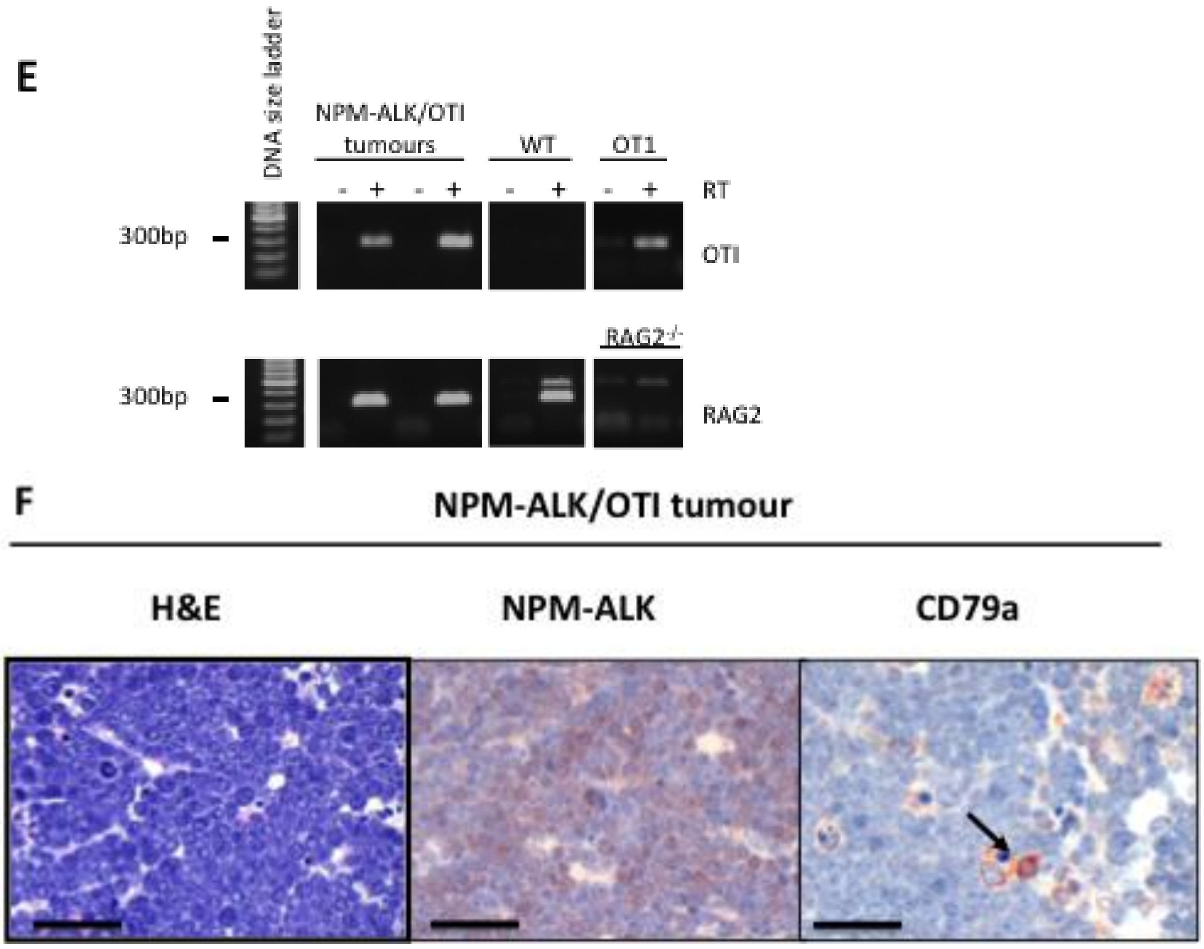

Supplementary Figure 8: T cell development is apparently normal in the thymi of NPM-ALK/OT1 transgenic mice and CD79a$^+$ B-cells are infrequent in the resulting tumours. (A) There is a significant increase in thymic cellularity in the NPM-ALK/OTI mice compared to OT1 littermate controls. $p<0.05$, Students t-test assuming equal variance, data represent the standard error of the mean (whiskers) and the mean (horizontal bars), NPM-ALK/OT1 n=4, OT1/WT n=2. MLN = mesenteric lymph nodes (B) The percentage of cells at each stage of thymic development are not significantly different at 6 weeks of age compared to OTI littermate control mice (no difference was observed at 10 weeks of age either – data not shown). The top two panels represent the double negative population alone whilst the lower panels are total live thymocytes. These data are representative of 3 mice analysed. (C) The number of T cells in the peripheral lymph nodes is also consistent between NPM-ALK and NPM-ALK/OT1 transgenic mice. These data are representative of 4 mice analysed. (D) Intracellular CD3 but not Vα2 (in red, isotype control in blue) is detected in tumour cells of NPM-ALK/OTI mice but not in unaffected lymph nodes. These data are representative of 3 mice analysed. (E) Transcripts for the OTI TCR transgene and RAG2 are detectable in the tumours of NPM-ALK/OTI transgenic mice. WT = wild-type mouse tail DNA, OTI = OTI transgenic mouse lymph node DNA, RAG$^{-/-}$ = RAG knockout mouse tail DNA. Two tumours are shown which are representative of at least 10 different NPM-ALK/OTI tumours. (F) A representative image is shown of 5 CD4NA/OTI tumours analysed by immunohistochemistry for cells expressing the B-cell marker CD79a. Scale bars represent 50 µm.

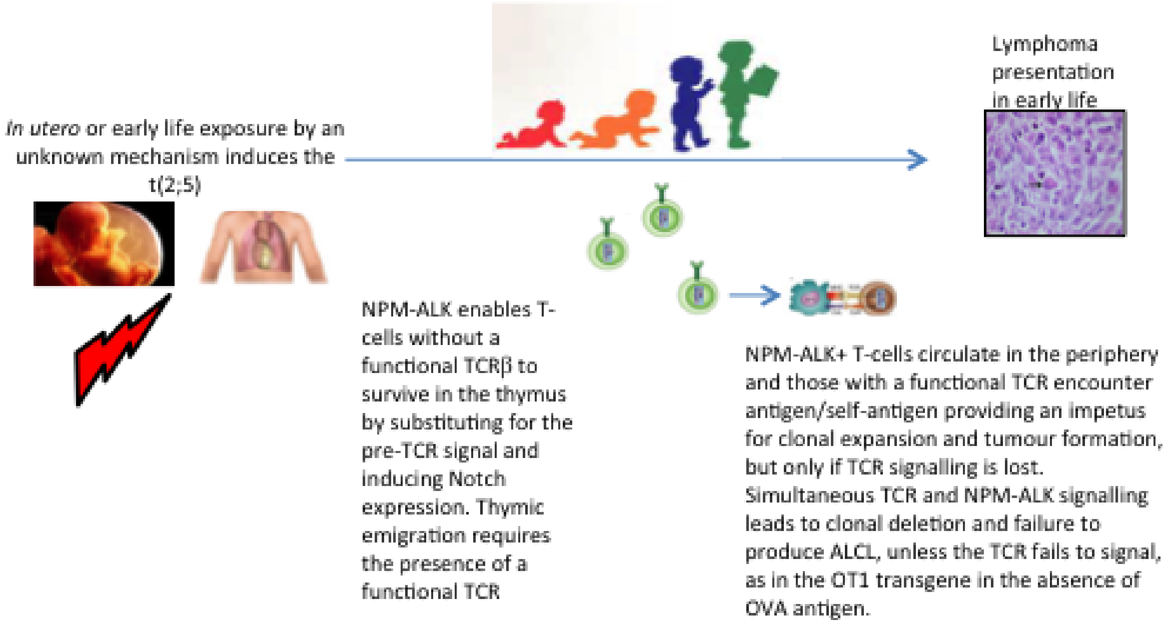

Supplementary Figure 9: Potential mechanism of NPM-ALK-induced lymphoma formation. The t(2;5) is present in the thymi of children and maybe even in haemopoietic progenitors as reported by (Laurent et al., 2012) resulting in subversion of thymic developmental processes. Thymocytes with aberrant TCR survive due to NPM-ALK induced replacement of the pre-TCR signal and expression of Notch 1. Hence, NPM-ALK enables thymocytes with incomplete/absent/non-functional TCR rearrangements to bypass the death signal. Both T cells with a normal TCR repertoire and those without then exit into the periphery in the latter case either through the presence of a TCR of sufficient format or by some other mechanism modulated by NPM-ALK. These aberrant T cells survive and circulate in the periphery in time undergoing full transformation with subsequent loss of expression of their TCR to become T cell lymphomas. This is consistent with the lack of a TCR on most ALCL despite the presence of some form of TCR rearrangement. It is possible that the pre-tumorigenic cells expressing NPM-ALK in the periphery mount an immune response dependent on their aberrant TCR (which could include self-antigens) and that subsequent loss of this TCR is permissive for transformation, an event that could be mediated by rapid proliferation subsequent to Ag recognition.

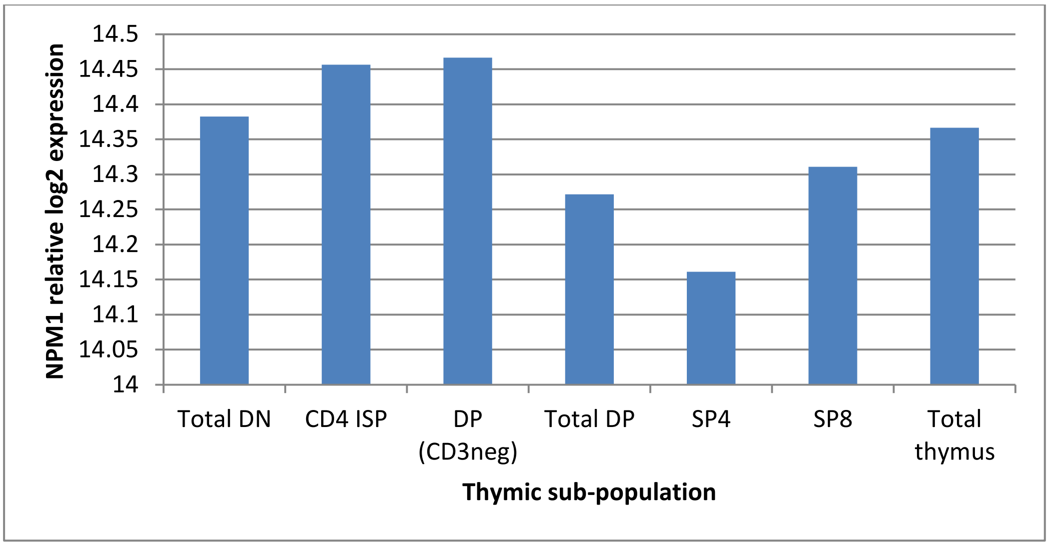

Supplementary Figure 10: NPM1 expression is constant in thymic sub-populations. NPM1 transcripts are present in thymic sup-populations isolated from human thymi. Data taken from GSE46170 of the NCBI GEO database, DP = $CD4^+CD8^+$ double positive, ISP = intermediate single positive, DN = double negative, SP = single positive, neg = negative. All data were analyzed in R with normalization using Quantile and log2 transformation

2a

18S

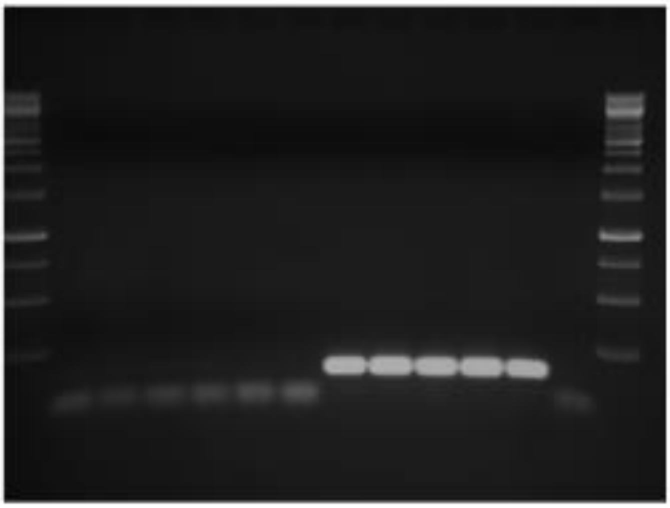

2a

NA

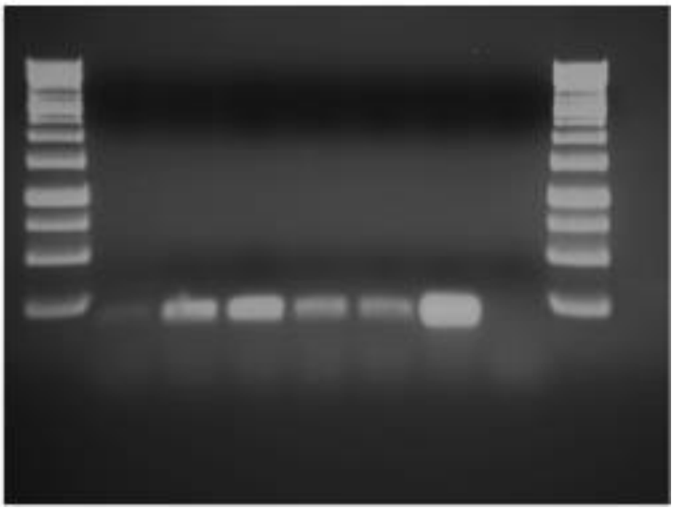

Supplementary Figure 11: Full gel images for Figure 2A

3c

TCR
NA/
RAG2

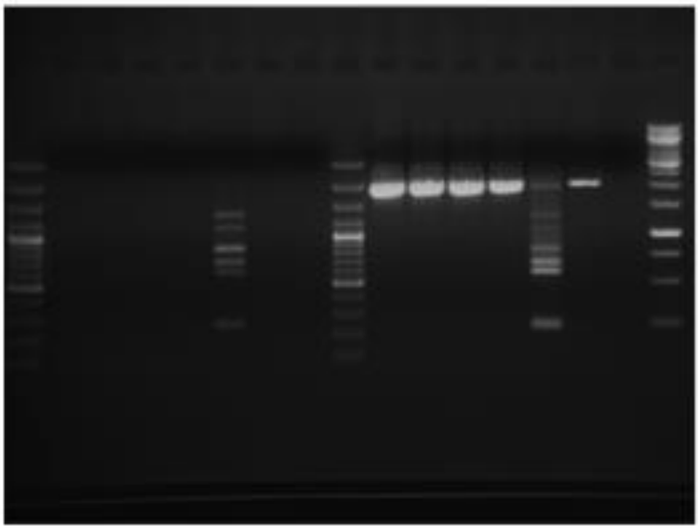

3c

TCR
NA

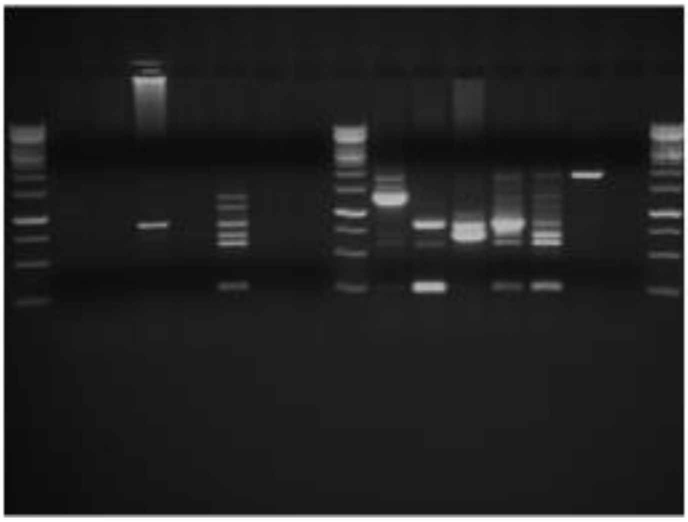

Supplementary Figure 12: Full gel images for Figure 3C

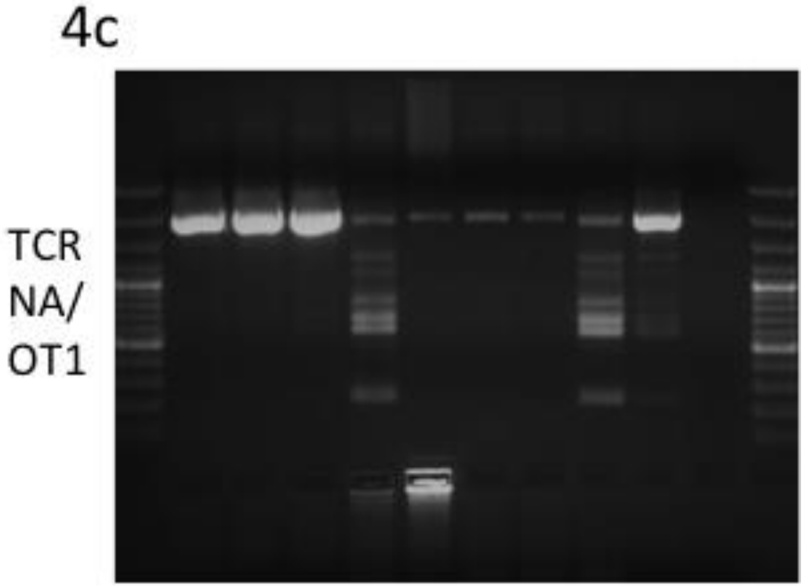

Supplementary Figure 13: Full gel image for Figure 4C

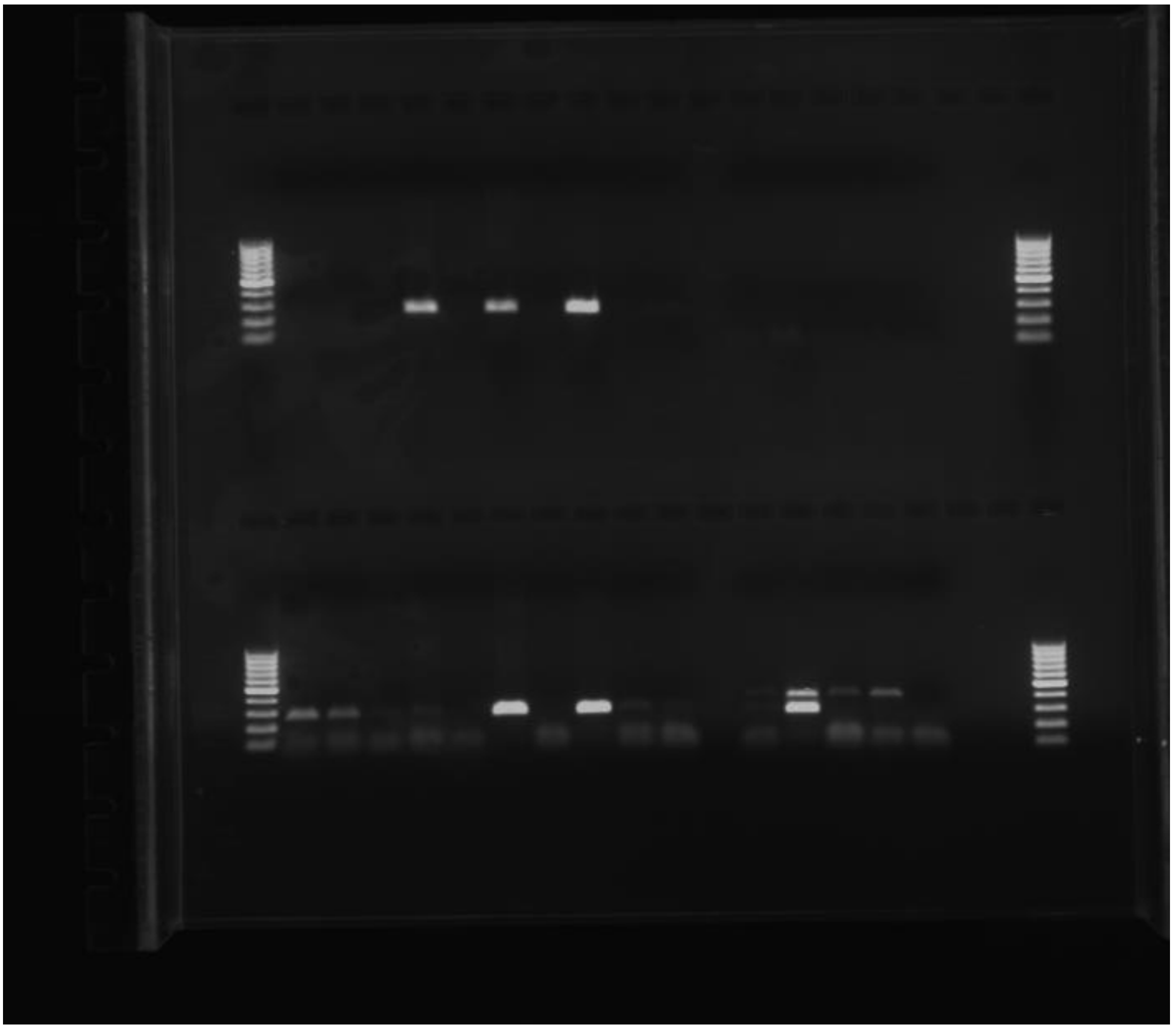

Supplementary Figure 14: Full gel image for Supplementary figure 6E

Supplementary Tables

ALCL UPN	Sex (M:1; F:2)	Adult or Paediatric	ALK partner	% infiltration*	CD2	CD3	CD4	CD5	CD7	CD8	Phenotype	TCRγ	TCRδ	TCRβ	TCRα transcript	TCRα/δ CGH	TCRβ CGH	TCR category
ALCL 37	2	P	NPM	70/50/60	1	0	1	1	0	0	T cyto	Neg	Neg	PCL	nd	GL	GL	GL
ALCL 20	1	P	NPM	60/50/50	0	0	1	0	0	0	T cyto	Neg	Neg	PCL	nd	GL	GL	GL
ALCL 25	1	P	NPM	70/70/70	1	0	1	0	1	0	T cyto	Neg	Neg	PCL	nd	GL	GL	GL
ALCL 11	2	P	NPM	70/70/70	0	0	0	1	1	0	T cyto	Neg	Neg	PCL	nd	GL	GL	GL
ALCL 74	2	A	NPM	60/60/60	nd	0	0	nd	nd	1	T cyto	Neg	Neg	PCL	nd	nd	nd	GL
ALCL 31	1	P	NPM	80/80/50	nd	0	0	0	nd	nd	null	Neg	Neg	PCL	nd	nd	nd	GL
ALCL 66	1	A	TPM3	60/70/60	0	0	0	0	0	0	null cyto	Neg	Neg	PCL	nd	nd	nd	GL
ALCL 77	2	A	NPM	60/50/40	1	0	1	0	0	0	T cyto	Neg	DD1-JD1<br>DD2-JD1	PCL	nd	nd	nd	GL
ALCL 27	2	P	NPM	50/50/50	1	0	0	0	1	0	T cyto	2 VJ<br>nd/nd	Neg	1VDJ<br>IF<br>1DJ	1VJ<br>IF	1 Va-Ja	GL	AB
ALCL 26	1	P	NPM	50/50/50	1	0	1	0	nd	0	T cyto	2 VJ<br>OF/OF	VD5-JA29 OF	2VDJ<br>OF/OF	1VJ<br>IF	1 Va-Ja	1VJ	AB
ALCL 24	2	P	NPM	80/80/80	nd	1	1	0	nd	0	T cyto	2 VJ<br>OF/OF	DD2-JD1	1VDJ<br>IF<br>1DJ	1VJ<br>IF	1 Va-Ja	GL	AB
ALCL 21	2	P	NPM	50/40/50	0	0	0	1	0	nd	T cyto	2 VJ<br>OF/OF	Neg	1VDJ<br>IF<br>2DJ	1VJ<br>IF	GL	GL	AB
ALCL 10	2	P	NPM	60/50/50	1	1	1	0	1	0	T cyto	2 VJ<br>nd/OF	Neg	1VDJ<br>IF<br>1DJ	1VJ<br>IF	1 Va-Ja	1VJ	AB
ALCL 1	1	P	NPM	80/70/70	nd	0	nd	1	nd	nd	T cyto	2 VJ<br>nd/OF	Neg	1VDJ<br>IF	1VJ<br>OF	1 Va-Ja	1VJ	AB
ALCL 29	1	P	NPM	70/70/70	0	0	1	0	0	0	T cyto	2 VJ	Neg	1VDJ<br>IF<br>1DJ	2VJ<br>Fail	1 Va-Ja	GL	AB
ALCL 39	1	P	NPM	80/80/80	1	1	0	0	1	1	T cyto	2 VJ<br>IF/nd	Neg	1VDJ<br>IF<br>1DJ	2VJ<br>IF/IF	1 Va-Ja	GL	AB
ALCL 19	1	P	NPM	60/60/60	1	0	1	1	1	0	T (cyto nd)	2 VJ<br>OF/OF	Neg	1VDJ<br>IF	2VJ<br>IF/IF	2 Va-Ja	GL	AB
ALCL 7	1	P	nd	70/90/0	1	0	0	0	0	0	T cyto	2 VJ<br>IF/OF	Neg	1VDJ<br>IF	2VJ<br>IF/OF	2 Va-Ja	GL	AB
ALCL 42	1	P	NPM	60/60/50	nd	0	nd	nd	nd	nd	nd	2 VJ	Neg	1VDJ	nd	nd	nd	AB
ALCL 40	2	P	TPM3	70/70/60	1	0	0	0	1	0	T cyto	2 VJ	Neg	1VDJ	nd	nd	nd	AB
ALCL 4	2	P	NPM	80/80/80	nd	0	nd	0	nd	1	T cyto	2 VJ	Neg	1VDJ<br>nd<br>1DJ	nd	1 Va-Ja	GL	AB
ALCL 38	1	P	NPM	50/50/40	1	0	2	1	0	1	T cyto	2 VJ	Neg	1VDJ<br>2DJ	nd	nd	nd	AB
ALCL	1	P	NPM	90/8	0	0	0	0	0	0	null	2 VJ	Neg	1VDJ	nd	1	GL	AB

12				0/80							cyto			IF 2DJ		Va-Ja		
ALCL 82	1	A	NPM	70	1	0	nd	0	0	nd	T (cyto nd)	2 VJ	Neg	1VDJ	nd	nd	nd	AB
ALCL 81	1	P	NPM	60/70/70	1	0	1	0	1	0	T cyto	2 VJ	Neg	1VDJ	nd	nd	nd	AB
ALCL 67	1	A	NPM	80/80/80	1	1	2	1	1	1	T cyto	2 VJ	Neg	1VDJ 1DJ	nd	nd	nd	AB
ALCL 64	1	A	NPM	70/50/70	1	1	2	1	nd	1	T cyto	2 VJ	Neg	1VDJ 1DJ	nd	nd	nd	AB
ALCL 62	1	P	NPM	90/90/90	0	0	0	1	0	0	T cyto	1 VJ	Neg	1VDJ 2DJ	nd	nd	nd	AB
ALCL 61	1	P	NPM	50/60/50	1	0	1	1	nd	0	T cyto	1 VJ	Neg	1VDJ	nd	nd	nd	AB
ALCL 60	2	P	NPM	70/70/70	1	0	0	1	nd	1	T cyto	2 VJ	Neg	1VDJ 1DJ	nd	nd	nd	AB
ALCL 59	1	P	NPM	60/60/50	nd	0	0	0	nd	0	T/null ? cyto	2 VJ	Neg	1VDJ 1DJ	nd	nd	nd	AB
ALCL 56	1	P	NPM	50/50/30	0	0	0	1	0	1	T cyto	2 VJ	Neg	1VDJ 1DJ	nd	nd	nd	AB
ALCL 55	2	P	NPM	90/90/90	0	1	0	0	1	1	T cyto	2 VJ	Neg	1VDJ 2DJ	nd	nd	nd	AB
ALCL 52	1	P	NPM	80/80/80	0	nd	0	0	0	1	T cyto	2 VJ	Neg	1VDJ 1DJ	nd	nd	nd	AB
ALCL 51	1	P	NPM	60/60/70	0	1	2	nd	1	2	T cyto	2 VJ	Neg	1VDJ	nd	nd	nd	AB
ALCL 6	1	P	NPM	60/80/30	0	0	0	0	0	1	T cyto	2 VJ	Neg	2VDJ IF/OF	PCL	1 Va-Ja	GL	AB
ALCL 5	1	P	NPM	80/80/80	nd	0	0	0	nd	1	T cyto	2 VJ	Neg	1VDJ IF	PCL	1 Va-Ja	GL	AB
ALCL 45	1	A	TPM 3	70/70/60	nd	nd	nd	nd	nd	nd	nd	2VJ	Neg	1VDJ IF 1DJ	PCL	1 Va-Ja	GL	AB
ALCL 35	2	P	NPM	60/60/60	1	0	1	0	1	0	T cyto	2 VJ	Neg	1VDJ IF	PCL	1 Va-Ja	GL	AB
ALCL 50	2	P	ATIC	80/70/ND	2	0	0	0	nt	1	T cyto	1 VJ	Neg	1VDJ IF 1DJ	PCL	1 Va-Ja	GL	AB
ALCL 33	2	P	NPM	80/80/80	1	1	2	1	1	nt	T cyto	2 VJ nd	VD3-JD1 IF DD2-JD1	1DJ	nd	1D dJd	GL	GD
ALCL 65	2	P	NPM	70/70/70	0	0	0	0	1	0	T cyto	2 VJ IF/nd	VD1-JD1 IF VD3-JD2 OF	1DJ	nd	nd	nd	GD
ALCL 17	2	P	ATIC	100/100/90	nd	nd	nd	nd	nd	nd	nd	2 VJ IF/OF	VD1-JD1 fail DD2-JD1	2DJ	nd	1 VdJd 1D dJd	GL	GD
ALCL 36	2	P	NPM	90/80/70	0	0	nd	0	nd	nd	null cyto	2 VJ IF/nd	VD1-JD1 fail DD2-JD1	1VDJ 1DJ	PCL	nd	nd	GD
ALCL 63	1	P	NPM	80/80/80	0	1	0	0	0	0	T (cyto nd)	2 VJ IF/OF	VD1-JD1 IF	1DJ	nd	nd	nd	GD
ALCL 18	1	A	NPM	70/60/60	nd	1	1	1	nd	0	T cyto	2 VJ IF/OF	VD1-JD1 IF DD2-JD1	1DJ	nd	1 VdJd 1D dJd	GL	GD
ALCL 9	1	P	NPM	80/70/70	1	0	nd	0	nd	nd	T cyto	2 VJ IF/OF	VD1-JD1 IF DD2-JD1	1DJ	nd	1 VdJd 1D dJd	GL	GD
ALCL	2	P	NPM	40/4	nd	1	2	0	1	0	T cyto	2 VJ	VD1-JD1	2DJ	nd	GL	GL	GD

22				0/40								IF/nd	IF DD2-JD1					
ALCL 57	2	A	NPM	50/60/60	1	0	2	0	nd	0	T cyto	2 VJ IF/OF	VD1-JD1 IF DD2-JD1	2DJ	nd	nd	nd	GD
ALCL 3	1	P	NPM	90/80/80	1	0	0	1	1	0	T cyto	2 VJ IF/OF	VD1-JD1 IF DD2-JD1	2DJ	nd	1 VdJd 1DdJd	GL	GD
ALCL 69	2	P	NPM	70/80/80	0	0	nd	0	0	nd	null	2 VJ IF/IF	VD1-JD1 IF DD2-JD1	1DJ	nd	nd	nd	GD
ALCL 30	2	P	NPM	70/70/80	nd	0	0	0	nd	0	null	2 VJ nd/nd	Neg	1DJ	1VJ IF	1 Va-Ja	GL	G -> TRA
ALCL 32	1	P	NPM	60/60/60	1	0	0	0	nd	1	T cyto	1 VJ OF	Neg	PCL	1VJ IF	1 Va-Ja	GL	G -> TRA
ALCL 2	1	P	NPM	60/40/30	1	0	1	1	nd	nd	T cyto	2 VJ nd/OF	Neg	1DJ	1VJ IF	GL	GL	G -> TRA
ALCL 53	1	P	NPM	60/60/50	1	0	2	1	0	0	T cyto	2 VJ OF/OF	Neg	PCL	2VJ IF/IF	nd	nd	G ->TRA
ALCL 43	1	P	TPM3	70/80/80	0	0	0	1	1	0	T cyto	2 VJ IF/nd	Neg	1DJ	1VJ IF	nd	nd	G -> TRA
ALCL 23	1	P	NPM	90	0	0	1	0	0	0	T cyto	1 VJ OF	Neg	PCL	PCL	GL	GL	G
**ALCL Cell Lines**																		
Karpas-299			NPM		0	1	1	1	0	1		2 VJ	Neg	1VDJ 1DJ	2VJ	nd	AB	nd
SU-DHL1			NPM		1	1	0	1	0	0		2 VJ	Neg	1VDJ	PCL	nd	AB	nd
COST			NPM		0	1	0	0	0	0		2 VJ	VD1-JD1	1DJ	PCL	nd	GD	nd

Supplementary Table 1: Overview of clinical, immunohistological and TCR analysis of ALCL cases. * Assessed from haematoxylin and eosin slides taken at the beginning, between and at the end of the blocks used for DNA and RNA extraction: nd = not determined; UPN = Unique patient number; GL = Germ line; T cyto = T cytotoxic; PCL = polyclonal. For the IHC analysis of cell surface protein expression (CD2, CD3, CD4, CD5, CD7 and CD8), 0=negative, 1=positive and 2 weak positive/doubtful.

	DN1	DN2	DN3	DN4	CD117 (DN)	Total thymocytes
CD4NA	$0.1 \times 10^6 \pm 0.02$	$0.1 \times 10^6 \pm 0.01$	$3.1 \times 10^6 \pm 0.32$	$1.3 \times 10^6 \pm 0.63$	$2.4 \times 10^6 \pm 0.29$	$158 \times 10^6 \pm 15$
Wild-type	$0.2 \times 10^6 \pm 0.03$	$0.1 \times 10^6 \pm 0.02$	$2.2 \times 10^6 \pm 0.21$	$0.9 \times 10^6 \pm 0.01$	$1.5 \times 10^6 \pm 0.10$	$143 \times 10^6 \pm 18$

Supplementary Table 2: Thymocyte sub-set numbers in the thymi of the indicated transgenic mouse genotypes. Absolute numbers ± s.e.m of the data presented in Figure 2C, n=5 for each genotype. Mice were 5 weeks of age.

	DN1	DN2	DN3	DN4	DP	CD4 SP	CD8 SP	Total thymocytes
CD4NA /RAG2$^{-/-}$	0.04 x 10^6 ± 0.01	0.02 x 10^6 ± 0.01	1.4 x 10^6 ± 0.48	0.29 x 10^6 ± 0.25	6.41 x 10^6 ± 0.22	2.11 x 10^6 ± 0.47	0.1 x 10^6 ± 0.01	10.3 x 10^6 ± 0.88
RAG2$^{-/-}$	0.04 x 10^6 ± 0.02	0.04 x 10^6 ± 0.02	3.0 x 10^6 ± 0.28	0.04 x 10^6 ± 0.01	0.03 x 10^6 ± 0.00	0.15 x 10^6 ± 0.03	0.04 x 10^6 ± 0.01	3.3 x 10^6 ± 0.33

Supplementary Table 3: Thymocyte sub-set numbers in the thymi of the indicated transgenic mouse genotypes. Absolute numbers ± s.e.m of the data presented in Figure 2E, n=10 for each genotype. Mice were 9 weeks of age.

NA/OTI/RAG2$^{-/-}$ (No MHV-Ova)			NA/OTI administered MHV-Ova		
Mouse	Survival (days)	Diagnosis	Mouse	Survival (days)	Diagnosis
1	231	Hepatocellular carcinoma	1	230	Hepatocellular carcinoma
2	250	GIST	2	123	Sarcoma
3	252	GIST	3	261	Hepatocellular carcinoma
4	131	Sarcoma	4	208	Lymphoma
5	346	Leiomyosarcoma	5	268	Hepatocellular carcinoma
6	93	GIST			
7	129	Hepatocellular carcinoma			
8	367	Sarcoma			
9	367	Sarcoma			
10	423	Hepatocellular carcinoma			

Supplementary Table 4: Tumour phenotypes detected in CD4NA/OTI/RAG2$^{-/-}$ and CD4NA/OTI mice. The left columns represent CD4NA/OTI/RAG2$^{-/-}$ (in the absence of MHV-ova administration) and the right columns NA/OTI mice administered MHV-ova.

	Primer Name	Sequence (5' - 3')
TCRβ	DB1	GCC-AAA-CAG-CCT-TAC-AAA-GAC
	DB2	TTT-CCA-AGC-CCC-ACA-CAG-TC
	Jbeta1-1_Fluo	joe_CTTACCTACAACTGTGAATCTGGTG
	Jbeta1-2_Fluo	joe_cttacctacaacggttaacctggtc
	Jbeta1-3_Fluo	joe_CTTACCTACAACAGTGAGCCAACTT
	Jbeta1-4_Fluo	joe_CATACCCAAGACAGAGAGCTGGGTTC
	Jbeta1-5_fluo	joe_CTTACCTAGGATGGAGAGTCGAGTC
	Jbeta1-6_fluo	joe_CATACCTGTCACAGTGAGCCTG

	Jbeta2-1_fluo	fam_CCTTCTTACCTAGCACGGTGA
	Jbeta2-2_fluo	fam_CTTACCCAGTACGGTCAGCCT
	Jbeta2-3_fluo	fam_CCCGCTTACCGAGCACTGTCA
	Jbeta2-4_fluo	fam_CCAGCTTACCCAGCACTGAGA
	Jbeta2-5_fluo	fam_CGCGCACACCGAGCAC
	Jbeta2-5_V2_fluo	fam_CGCGCTCAC-CGA-GCA-C
	Jbeta2-6_fluo	fam_CTCGCCCAGCACGGTCAGCCT
	Jbeta2-7_fluo	famCTTACCTGTAACCGTGAGCCTG
	VB10	TTATGTTTACTGGTATCGTAAGAAGC
	VB11	CAAAATGTACTGGTATCAACAA
	VB13A	ATACATGTACTGGTATCGACAAGAC
	VB13B	GGCCATGTACTGGTATAGACAAG
	VB14	GTATATGTCCTGGTATCGACAAGA
	VB16	TAACCTTTATTGGTATCGACGTGT
	VB17	GGCCATGTACTGGTACCGACA
	VB18	TCATGTTTACTGGTATCGGCAG
	VB19	TTATGTTTATTGGTATCAACAGAATCA

	VB2	AACTATGTTTTGGTATCGTCA
	VB20	CAACCTATACTGGTACCGACA
	VB21	TACCCTTTACTGGTACCGGCAG
	VB22	ATACTTCTATTGGTACAGAGACAAATCT
	VB23	CACGGTCTACTGGTACCAGCA
	VB24	CGTCATGTACTGGTACCAGCA
	VB4	CACGATGTTCTGGTACCGTCAGCA
	VB5	CAGTGTGTCCTGGTACCAACAG
	VB6A	AACCCTTTATTGGTACCGACA
	VB6B	ATCCCTTTTTTGGTACCAACAG
	VB6C	AACCCTTTATTGGTATCAACAG
	VB7A	CGCTATGTATTGGTACAAGCA
	VB8A	CTCCCGTTTTCTGGTACAGACAGAC
	VB9	CGCTATGTATTGGTATAAACAG
TCRδ	VD8	CAGCCAAATCCTTCAGTCTCAA
	VD6	CCCTGCATTATTGATAGCCAT
	VD5	ACCCTGCTGAAGGTCCTACAT

	VD4	ATGACCAGCAAAATGCAACAG
	VD3	GTACCGGATAAGGCCAGATTA
	VD2_fluo_vic 3130	(vic)ATACCGAgAAAAggACATCTATg
	VD1_fluo_ned 3130	(ned)ATgCAAAAAgTggTCgCTATT
	VD1	ATGCAAAAAGTGGTCGCTATT
	JD4	TTGTACCTCCAGATAGGTTCC
	JD3	CTCACGGGGCTCCACGAAGAG
	JD2	GTTCCACGATGAGTTGTGTTC
	JD1	GTTCCACAGTCACACGGGTTC
	Jalpha29	GGC AAA AGC ATT CTA GGT ACA
	DD3_3	TGGGACCCAGGGTGAGGATAT
	DD2-5'_fluo_fam 3130	(6fam)AgCgggTggTgATggCAAAgT
TCRα	VA1-1	GAGCCATTGTCCAGATAAACTG
	VA12-1	CCAGAGGGAGCCACTGTC
	VA12-2	GAGCCATTGCCTCTCTCAACT
	VA12-3	GGGAGCCATTGTTTCTCTCA
	VA30	GGGGAAGATGCTGTCATCAA

	VA35	GGAGAAGATGTCTCCATGAACT
	VA39	CATGCAGGAGGGAAAAAACT
	VA40	GGAGGGAGCATCTGTGACT
	VA41	GCCCAGGAAGGAGAATTTATCA
	VA8-4	GGAGCCCTGGTTCTGCTG
	VA8-7	AAGAAGCCCCTCTGGAACTG
	VA13-1	GAGACAGCGCTGTTATCAAGTG
	VA16	TTTAAAGGGGCCCCAGTG
	VA17	AGGAGGGTGAAAATGCCA
	VA18	CCAGTTACCCTCCCTGAGAG
	VA3	GTTGCTGAAGGGAATCCTCT
	VA31	GTGAGACCGTGAAACTGGACT
	VA33	AGGCAGAAAGGAGTAGCTGTGA
	VA38-2	TCTGTGCAGGAGGCAGAGA
	VA4	AGAAGTGAACATAACCTGTAGCCA
	VA9-2	AAACTGCACGTACACAGCCA
	VA22	GGAGGGAGCCAATTCCAC

	VA24	GGGAGACAGCACCAATTTCA
	VA26-2	GAGCCTGTTCACTTGCCTTG
	VA27	GGGAGAAAATCTCACTGTGTACT
	VA29	GCGTCCAGGAAGGAAGAATTT
	VA32	GAAATGGCCGTTATTAATGACA
	VA36	GGGAGACACCGTAACTCTCAAT
	VA37	GGAAGGTGACAGCGTCACA
	VA38-1	GCAGGAGGCAGAGACTGTG
	VA5	ACAGCTCCGTTATAAACTGCAC
	VA8-1	GCCTCACTGGAGTTGGGAT
	VA19	TGGTGGAGAAGGAGGATGTG
	VA2	GGAGCTGTGGTGGAAATCT
	VA20	CAGGAGGGAGAGAGTAGCAGTC
	VA23	CCAGAAAGGAGGGATTTCAATTA
	VA26-1	GCTGCAAACCTGCCTTGTAA
	VA28	GAAGAAATTCATTCCTGGTGTG
	VA6	ACATTCAGGAGGGTAAAACGG

	VA7	CGTTGCCTCCATGAGCTG
	VA8-3	GGAGCCTCACTGGAGTTGAGA
	VA8-5	GAAGGAGCCTCACTGGAGTT
	VA8-6	AAGCCCCTGTGGAGCTG
	VA10	GGAAAGAACTGCACTCTTCAA
	VA11	GGAATGCATGCCGTTCTTAAT
	VA1-2	GTGCCATTGTCCAGATCAACT
	VA13-2	CCAGGAGGGTGACAACTCTATT
	VA14	AGGAGGCTGTGACTCTGGACT
	VA15	GGGAATGCACAACATTCTTAAT
	VA21	AAGGAGAAAACTTGGTTCTCAACT
	VA25	GGAGAGGACTTCACCACGTACT
	VA34	GGGAAAGAATCTCACCATAAACT
	VA8-2	AACCCCGGTGCTGCTG
	VA9-1	TTCCCTGATTGTGAACTGCTC
	CA_Fluo	Ned_GGTACACGGCAGGGTCAG
	VA1-1	GAGCCATTGTCCAGATAAACTG

	VA12-1	CCAGAGGGAGCCACTGTC
	VA12-2	GAGCCATTGCCTCTCTCAACT
	VA12-3	GGGAGCCATTGTTTCTCTCA
	VA30	GGGGAAGATGCTGTCATCAA
	VA35	GGAGAAGATGTCTCCATGAACT
	VA39	CATGCAGGAGGGAAAAAACT
	VA40	GGAGGGAGCATCTGTGACT
	VA41	GCCCAGGAAGGAGAATTTATCA
	VA8-4	GGAGCCCTGGTTCTGCTG
	VA8-7	AAGAAGCCCCTCTGGAACTG
	VA13-1	GAGACAGCGCTGTTATCAAGTG
	VA16	TTTAAAGGGGCCCCAGTG
	VA17	AGGAGGGTGAAAATGCCA
	VA18	CCAGTTACCCTCCCTGAGAG
	VA3	GTTGCTGAAGGGAATCCTCT
	VA31	GTGAGACCGTGAAACTGGACT
	VA33	AGGCAGAAAGGAGTAGCTGTGA

	VA38-2	TCTGTGCAGGAGGCAGAGA
	VA4	AGAAGTGAACATAACCTGTAGCCA
	VA9-2	AAACTGCACGTACACAGCCA
	VA22	GGAGGGAGCCAATTCCAC
	VA24	GGGAGACAGCACCAATTTCA
	VA26-2	GAGCCTGTTCACTTGCCTTG
	VA27	GGGAGAAAATCTCACTGTGTACT
	VA29	GCGTCCAGGAAGGAAGAATTT
	VA32	GAAATGGCCGTTATTAATGACA
	VA36	GGGAGACACCGTAACTCTCAAT
	VA37	GGAAGGTGACAGCGTCACA
	VA38-1	GCAGGAGGCAGAGACTGTG
	VA5	ACAGCTCCGTTATAAACTGCAC
	VA8-1	GCCTCACTGGAGTTGGGAT
	VA19	TGGTGGAGAAGGAGGATGTG
	VA2	GGAGCTGTGGTGGAAATCT
	VA20	CAGGAGGGAGAGAGTAGCAGTC

	VA23	CCAGAAAGGAGGGATTTCAATTA
	VA26-1	GCTGCAAACCTGCCTTGTAA
	VA28	GAAGAAATTCATTCCTGGTGTG
	VA6	ACATTCAGGAGGGTAAAACGG
	VA7	CGTTGCCTCCATGAGCTG
	VA8-3	GGAGCCTCACTGGAGTTGAGA
	VA8-5	GAAGGAGCCTCACTGGAGTT
	VA8-6	AAGCCCCTGTGGAGCTG
	VA10	GGAAAGAACTGCACTCTTCAA
	VA11	GGAATGCATGCCGTTCTTAAT
	VA1-2	GTGCCATTGTCCAGATCAACT
	VA13-2	CCAGGAGGGTGACAACTCTATT
	VA14	AGGAGGCTGTGACTCTGGACT
	VA15	GGGAATGCACAACATTCTTAAT
	VA21	AAGGAGAAAACTTGGTTCTCAACT
	VA25	GGAGAGGACTTCACCACGTACT
	VA34	GGGAAAGAATCTCACCATAAACT

	VA8-2	AACCCCGGTGCTGCTG
	VA9-1	TTCCCTGATTGTGAACTGCTC
	CA_Fluo	Ned_GGTACACGGCAGGGTCAG
TCRγ	Jg1/2_ext	TACCTgTgACAACCAgTgTTg
	Jg1/2_fluo_vic 3130	(vic)TACCTgTgACAACCAgTgTTg
	Vg11	CTTCCACTTCCACTTTGAAA
	JgP_fluo_ned 3130	(ned)ACTTACCTgTAATgATAAgCTTT
	JgP1/2_fluo_fam 3130	(6fam)TTACCAggCgAAgTTACTATg
	JgP_ext	ACTTACctgtaatgataagcttt
	Vg9_2	ACCTGGTGAAGTCATACAGTTC
	VgF1	ACT-GGT-ACC-TAC-ACC-AGG-AGG
	JgP1/2_ext	TTA-CCA-GGC-GAA-GTT-ACT-ATG
	Vg10-2	AGC-ATG-GGT-AAG-ACA-AGC-AA
	Jg1/2_ext	TACCTgTgACAACCAgTgTTg
	Vg10-2	AGC-ATG-GGT-AAG-ACA-AGC-AA

Supplementary Table 5: Primers employed for Genescan analysis of human TCR rearrangements
